# Supplementary material for: Differential Regulation of G1 CDK Complexes by the Hsp90-Cdc37 Chaperone System
Source: Cell Rep. 2017 Oct 31;21(5):1386–98. doi: 10.1016/j.celrep.2017.10.042 (PMC5681435; doi:10.1016/j.celrep.2017.10.042)
Supplement: Document S1. Supplemental Experimental Procedures and Figures S1–S4 [file mmc1.pdf]

Cell Reports, Volume *21*

Supplemental Information

Differential Regulation of G1 CDK Complexes by the Hsp90-Cdc37 Chaperone System

Stephen T. Hallett, Martyna W. Pastok, R. Marc L. Morgan, Anita Wittner, Katie L.I.M. Blundell, Ildiko Felletar, Stephen R. Wedge, Chrisostomos Prodromou, Martin E.M. Noble, Laurence H. Pearl, and Jane A. Endicott

Supplemental Figures

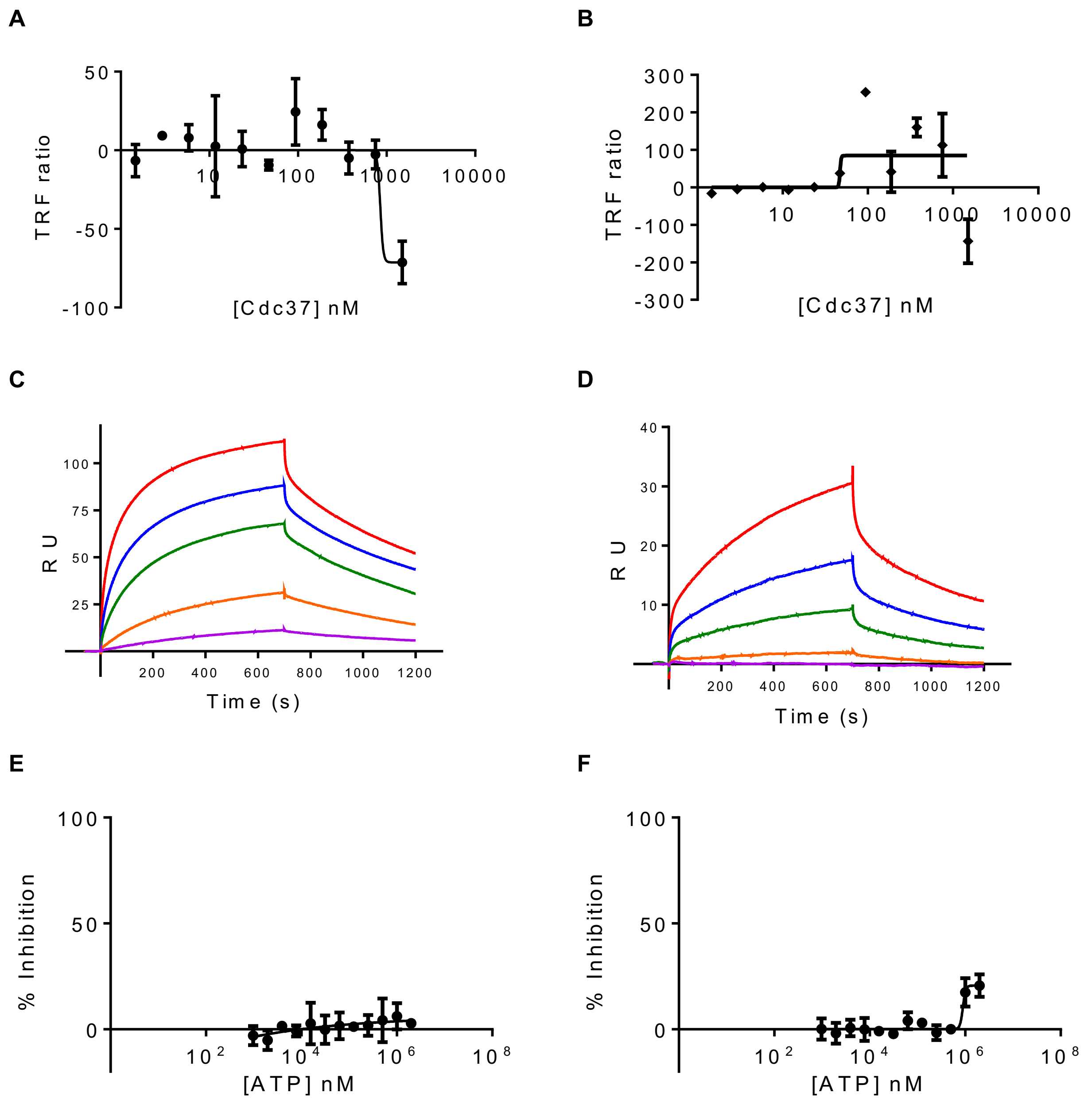

Figure S1. Related to Figure 1. Evaluation of CDK binding to Cdc37 and ATP. (A) CDK2 binding to Cdc37. The binding of full length authentic Cdc37 to CDK2 was tested by homogeneous time resolved fluorescence**.** Cdc37 does not bind to CDK2. The glutathione-S-transferase-CDK2 (GSTCDK2) concentration was 25 nM and measurements were carried out in duplicate. (**B**) Cdc37 does not bind to GST. The experiment in panel (A) was repeated replacing GSTCDK2 with GST (25 nM). In both panels, the error bars indicate standard deviation. (**C, D**) CDK4 (**C**) and CDK6 (**D**) binding to Cdc37 as measured by surface plasmon resonance. GSTCDK4 and GSTCDK6 were immobilized on the chip via anti-GST antibody coupling and the Cdc37 ligand was assayed in triplicate over a 5-point serial dilution (4000 nM, 2000 nM, 1000 nM, 250 nM and 62.5 nM). Dissociation constants (CDK4, $K_d = 347 \pm 30$ nM; and CDK6, $K_d = 7000 \pm 4500$ nM) were derived by using the Biacore S200 Evaluation Software. (**E, F**) ATP does not displace Cdc37 from CDK4 (**E**) or CDK6 (**F**). The ATP was dispensed in a two-fold dilution series starting at 2 mM. DMSO was added at 0.5% (**E**) or 0.01% (**F**). The concentrations of GSTCDK4 and GSTCDK6 were 8 nM and 6 nM respectively.

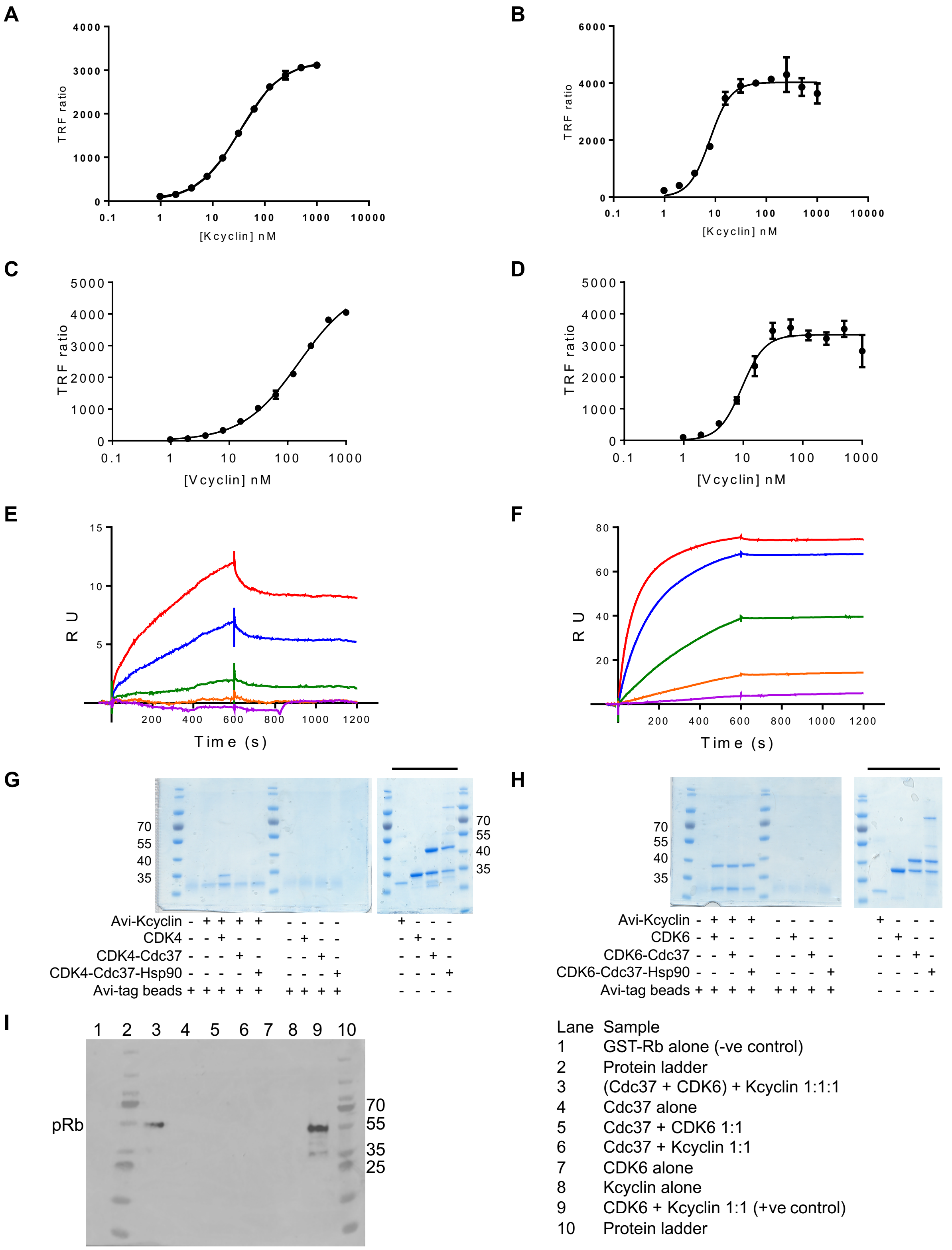

Figure S2. Related to Figure 2.

Kcyclin and Vcyclin bind to CDK4 and CDK6. (**A-D**) Homogeneous Time Resolved Fluorescence (HTRF) to measure the binding of Kcyclin (**A, B**) and Vcyclin (**C, D**) to CDK4 (**A, C**) and CDK6 (**B, D**). The concentrations of GSTCDK4 and GSTCDK6 were 10 nM. HTRF measurements were carried out in duplicate and repeated twice on two separate days. The error bars indicate standard deviation. (**E, F**) Surface Plasmon Resonance (SPR) to measure the binding of Vcyclin to CDK4 (**E**) and CDK6 (**F**). GSTCDK4 and GSTCDK6 were immobilized on the chip via anti-GST antibody coupling and the Vcyclin ligand was assayed in triplicate over a 5 point serial dilution (500 nM, 250 nM, 62.5 nM, 15.6 nM and 4 nM). Curves were evaluated using the Biacore S200 Evaluation Software. Although binding was detectable between CDK4 and Vcyclin (**E**), the data quality precluded calculation of a K_d. The dissociation constant for the interaction between CDK6 and Vcyclin ($K_d = 0.016 \pm 0.013$ nM) was derived by using the Biacore S200 Evaluation Software. **(G, H)** SDS-PAGE gels to accompany Figure 2E, F to confirm that CDK4 and CDK6 do not bind non-specifically to Avi-beads. Bars above the gels indicate inputs. Samples were visualized by InstantBlue staining. **(I)** CDK6-Kcyclin assembled following Kcyclin displacement of CDK6 from CDK6-Cdc37 is catalytically active. CDK6 activity was measured against retinoblastoma protein (pRb). A full description of the kinase assay is included in the Supplemental Experimental Procedures. Phosphorylated pRb was detected by an anti-phosphorylated S780 Ab (Abcam).

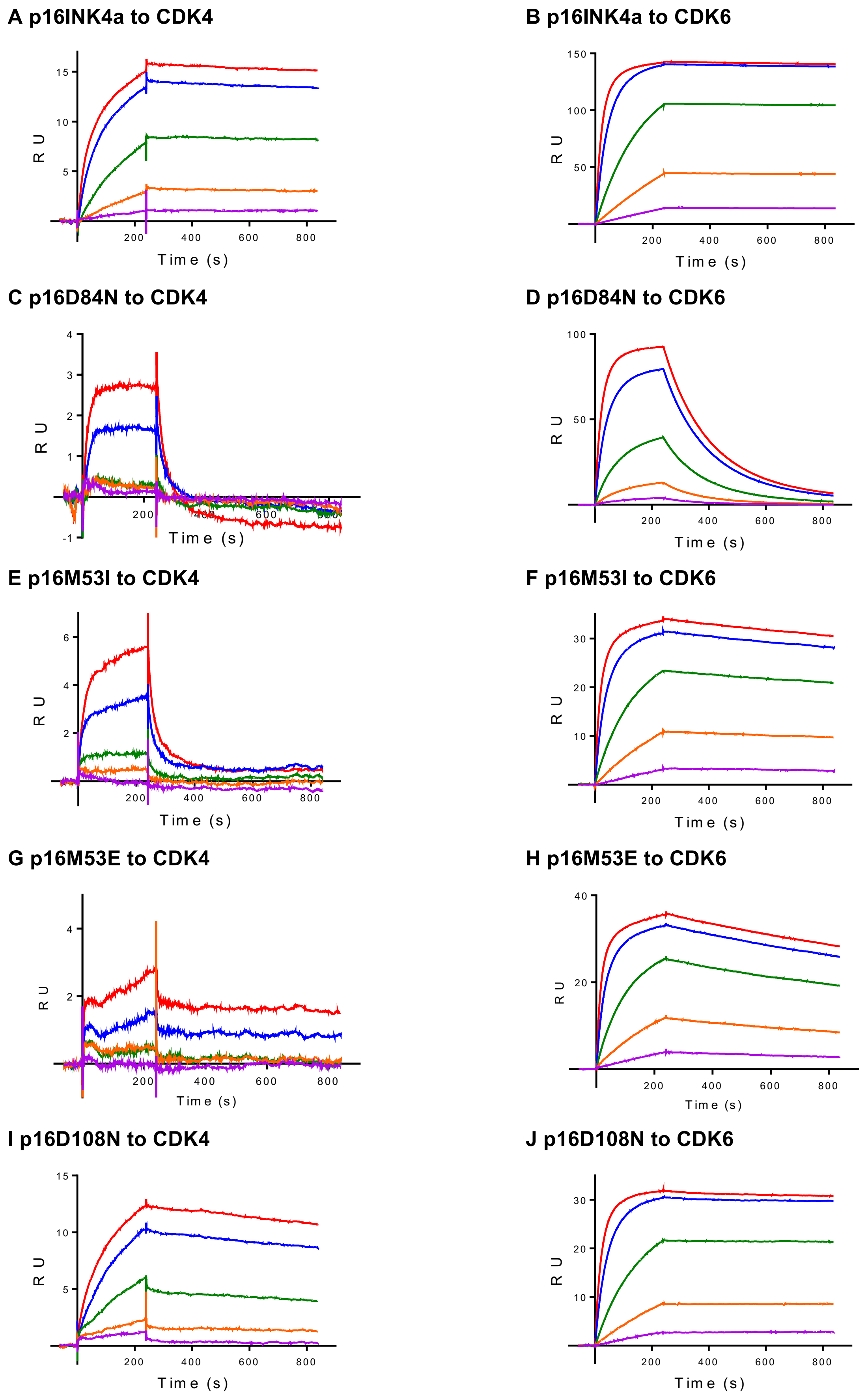

Figure S3. Related to Figure 3. p16INK4a binding to CDK4 and CDK6 measured by surface plasmon resonance. Binding of p16INK4a and p16INK4a mutants to CDK4 (**A, C, E, G, I**) and CDK6 (**B, D, F, H, J**) by SPR. GSTCDK4 or GSTCDK6 were immobilized on the chip via anti-GST antibody coupling and p16INK4a proteins were flowed over in a 5-point dilution series (200nM, 100 nM, 25 nM, 6.25 nM, and 1.56 nM). K_d values were derived using the Biacore S200 Evaluation Software and are presented in **Table 2**.

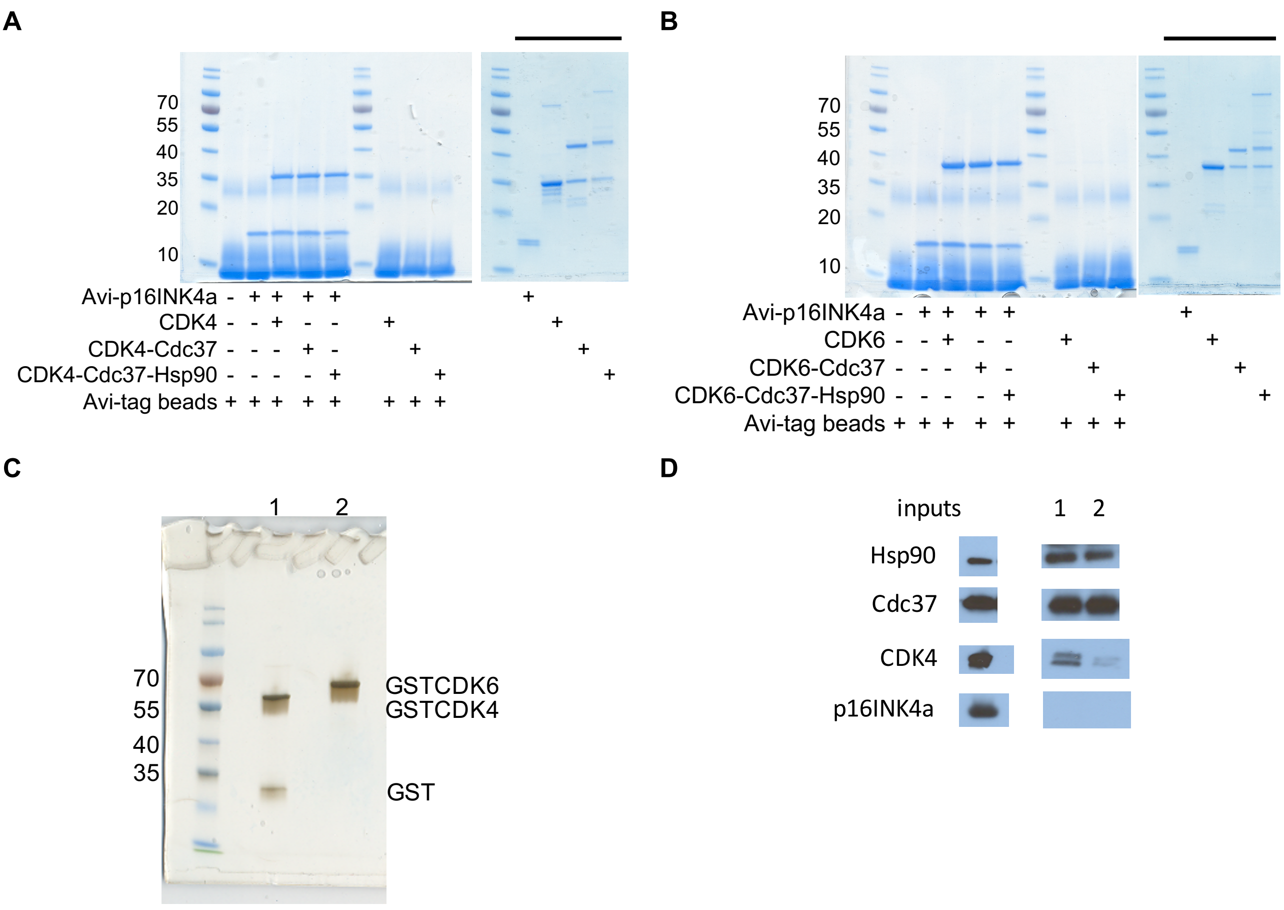

Figure S4. Related to Figure 3 and Experimental methods. Characterization of CDK4 and CDK6. (A, B) CDK4 and CDK6 do not bind non-specifically to Avi-tagged beads. Uncropped SDS-PAGE gels to accompany Figure 3 to confirm there is no non-specific binding of CDK4 and CDK6 to Avi-tag-beads. Bars above the gels indicate inputs. **(A)** CDK4, **(B)** CDK6. Samples were analyzed by SDS-PAGE and visualized by InstantBlue staining. **(C, D) Generation of GSTCDK4 and GSTCDK6 complexes from insect cells. (C)** Silver stained SDS-PAGE gel of purified GSTCDK4 and GSTCDK6. GSTCDK samples used in the HTRF assay were analyzed by SDS-PAGE and subsequent silver-staining. Lane 1, GSTCDK4; lane 2, GSTCDK6. The lower band in the GST-CDK4 sample is a small amount of contaminating GST. **(D)** The SfHsp90-Cdc37-CDK4 complex is disrupted by p16INK4a. SfHsp90-Cdc37-CDK4 purified from insect cells was incubated with buffer (lane 1) or p16INK4a (lane2) and immuno-precipitated with anti-Hsp90 antibodies. Subsequent to SDS-PAGE samples were western blotted using anti -Hsp90, -Cdc37, -CDK4 and -p16INK4a antibodies. CDK4 forms a stable complex with Hsp90 and Cdc37 (lane 1) but is displaced from Hsp90 upon addition of p16INK4a (lane 2). Cdc37 remains associated with Hsp90 upon client kinase displacement (lane 2). Input samples are 10% input.

Supplemental Experimental Procedures

Materials and reagents

Terbium-labelled anti-GST antibody and streptavidin-labelled XL665 dye were purchased from Life Technologies and Cisbio, respectively. CDK4/6 inhibitors PD0332991 (palbociclib) was purchased from Sigma Aldrich, and LY2835219 (abemaciclib) and LEE011 (ribociclib) from MedChem Express, and the Hsp90 ATP-competitive inhibitor NVP-AUY922 was supplied by Santa Cruz Biotechnology. Baculovirus expression for protein production in insect cells *Sf*9 was performed using the MultiBac™ system (Geneva-Biotech).

Protein expression

Human CDK4 and CDK6 were expressed in *Sf*9 insect cells using a recombinant baculovirus expression system (Bieniossek et al., 2012), Geneva-Biotech MultiBac™). Full length human CDK4 and CDK6 with an N-terminal glutathione-S-transferase (GST)-tag and 3C protease recognition site, and human untagged Cdc37$_{(1\text{-}348)}$ were cloned separately into the pACEBac1 acceptor vector. Human untagged full-length cyclin D1 and cyclin D3 were cloned into the pIDK donor vector. *Cre* recombinase (New England Biolabs) was used in the *Cre-Lox*P reaction of acceptor and donor vectors to generate the multigene fusion for co-expression of CDK4 or CDK6 with either cyclin D1 or cyclin D3. All constructs were verified by restriction enzyme digestion and DNA sequencing. Approximately 5-10 ng of DNA vector (either pACE-BAC-1-CDK, pACE-BAC-1-Cdc37 or an acceptor-donor fusion pACE-Bac-1-pIDK for CDK and cyclin D co-expression) was used to transform *EmBacY E. coli* cells harboring the EMBacY MultiBac™ bacmid (for constitutive expression of yellow fluorescent protein YFP). DNA sequences of interest were transferred to the bacmid via transposition into the mini Tn7 attachment site. White recombinant colonies were selected for subsequent bacmid preparation. MultiBac bacmid DNA was prepared by alkaline lysis (QIAprep Miniprep kit (Qiagen)). The final supernatant was precipitated using isopropanol (40%) and the resulting pellet was then washed twice with 70% ethanol, dried and re-suspended in sterilised water in sterile conditions. For the transfection reaction, transfection reagent (Gene Juice™ (Novagen)) was added to the re-suspended bacmid DNA and the resulting cocktail was used to infect $0.5*10^6$ *Sf9* cells seeded in 6-well plates. After 48-60 hr incubation at 27 ^{o}C, the supernatant was collected and positive transfection was verified by monitoring the appearance of yellow cells constitutively expressing the YFP gene and also containing the constructs of interest by fluorescence microscopy. This initial virus stock (Vo) was amplified twice and then used for protein expression.

Other proteins were expressed from recombinant *E. coli* cells. Human CDK2 was expressed as a GST fusion from the pGEX6P-1 vector backbone (GE Healthcare) as previously described (Brown et al., 1999). The full-length DNA sequences encoding herpesvirus saimiri cyclin (Vcyclin) and Kaposi's sarcoma-associated herpesvirus cyclin (Kcyclin) were synthesized (Integrated DNA Technologies). These sequences were sub-cloned into pGEX6P-1 to generate N-terminal GST fusion proteins and also further modified to include either a C-terminal Avi or FLAG tag. Mutant p16INK4a sequences were generated by site-directed mutagenesis using the QuikChange method (Agilent Technologies) or by gene synthesis (Integrated DNA Technologies) and verified by sequencing. The INK sequences were subsequently cloned into a modified pET3d vector to produce a N-terminal His$_6$-tagged protein that upon 3C cleavage generates the INK modified by a N-terminal Avi-tag. Full-length p27KIP1, p27KIP1, p21CIP1 and pRb fragments were expressed as GST-fusions from a pGEX6P-1 backbone. For the pull down experiments, full-length Cdc37 was modified with a N-terminal 3C protease-cleavable hexa-histidine tag following cloning into a pRSETA vector backbone. 3C cleavage generates a Cdc37 construct with a FLAG-tag at the N-terminus. For the HTRF experiments, Cdc37 was expressed from a modified pET3d vector to generate C-terminally Avi-tagged proteins. The Hsp90β full length construct was used in this study. This sequence was also expressed with an N-terminal 3C protease-cleavable hexa-histidine tag from a pRSETA vector backbone and for the HTRF assay was expressed from a modified pET3d vector backbone to generate an N-terminally Avi-tagged protein.

Protein purification

Insect cells were harvested after 72 hours of infection, re-suspended in 10 mM HEPES pH 7.5, 150 mM NaCl, 10% glycerol, 0.5 mM EDTA, 0.5 mM TCEP (resuspension buffer) supplemented with a protease inhibitor cocktail (Roche) and stored at -20 ^{o}C. Cell pellets were sonicated on ice followed by centrifugation at 48254xg for 1 hr at 4 ^{o}C. The filtered supernatant was incubated with 1 ml of glutathione Sepharose (GE Healthcare) for 3 hr on a rocker at 4 ^{o}C. The Sepharose resin was then loaded into a column and exhaustively washed with 30 ml of resuspension buffer. Bound proteins were eluted by 20 mM gluthathione at pH 8.0, cleaved overnight with 3C protease (1:50 w/w) and then further purified using size exclusion chromatography (SEC) (HiLoad 16/60 Superdex 75 pg for proteins smaller than ~60 kDa or a 200 pg for proteins or complexes larger than ~60 kDa). To ensure removal of any contaminating GST dimer, appropriate SEC fractions were pooled, incubated with glutathione Sepharose and then concentrated.

Human CDK2, INKs, p21CIP1, p27KIP1, Cdc37, Hsp90β and viral cyclins were expressed in *E. coli* strain BL21STAR(DE3) (Life Technologies) (CDK2, INKs, p21CIP1, p27KIP1, Cdc37, and viral cyclins) or Rosetta (DE3) (Cdc37 and Hsp90β). Briefly, recombinant *E. coli* BL21STAR(DE3) cells were grown at 30 ^{o}C in 2xYT medium till OD_{600}~0.7-0.8, induced at 18 ^{o}C by adding 0.5 mM IPTG and further incubated for 16 hr. Alternatively recombinant *E. coli* Rosetta (DE3) cells (Merck Millipore) expressing Cdc37 or Hsp90β were grown at 37 ^{o}C in LB medium till the OD_{600} reached ~0.2 and 0.6 respectively. The cells were then induced at 18 ^{o}C by adding 0.5 mM IPTG and incubated for 16 hr. GST-tagged proteins were first purified by an affinity chromatography step, followed by 3C protease cleavage to remove the GST tag and then subsequent SEC. If required, appropriate SEC fractions were pooled and re-applied to a glutathione column to remove any contaminating GST and the flow-through collected. His$_6$-tagged proteins were purified using Ni-NTA His-Trap (GE Healthcare), cleaved and further purified using sequential SEC and ion

exchange chromatography (HiTrap Q, GE Healthcare). Protein concentrations were determined by NanoDrop2000 UV-Vis Spectrophotometer (Thermo Scientific) or using a Shimadzu UV1800 spectrophotometer. A representative SDS-polyacrylamide gel stained with silver is shown in Figure S4 to illustrate the purity of the GSTCDK4 and GSTCDK6 post purification.

Protein biotinylation
Avi-tagged proteins (Cdc37, p16INK4a, p16INK4a mutants, Kcyclin and Vcyclin) were biotinylated to provide an affinity tag for the streptavidin-tagged XL665 dye. A substantial amount of biotinylated protein was purified from the recombinant *E. coli* cells as a result of endogenous BirA activity. Proteins were further modified by incubating 40 µM of the Avi-tagged protein with 10 µg BirA in biotinylation buffer (50 mM Bicine pH 8.3, 10 mM ATP, 10 mM MgOAc, 50 µM d-biotin) at 30°C for 60 minutes. Protein was then buffer exchanged into HTRF buffer A (50 mM HEPES pH 7.5, 100 mM NaCl, 0.1 mg/ml BSA, 1 mM DTT) using a PD-10 desalting column (GE Healthcare). The extent of protein biotinylation was monitored by pull downs using High Capacity NeutrAvidin® Agarose Resin (Thermo Scientific Pierce).

Pull-down assays
For the pull down assays presented in Figure 2C, D, 6.3 µg (0.12 nmol) of FLAG-tagged Cdc37 was incubated for 1 hr at 4 ^{o}C with 25 µl anti-FLAG M2 agarose beads in a total volume of 60 µl in 20 mM Tris pH 7.5, 150 mM NaCl, 5 mM Na_2MoO_4, 0.5 mM EDTA, 0.5 mM TCEP, and 0.5% Igepal. Using this buffer non-specific protein binding to the beads was minimized. A 3-fold molar excess (0.36 nmol) of the second protein (CDK or CDK-cyclin D was then added and incubation continued for a further hour at 4 ^{o}C. Each pull down was washed three times with 200 µl of buffer and then 15 µl of SDS-PAGE sample buffer was added. Boiled and spun down samples were subjected to SDS-PAGE and proteins visualized by InstantBlue staining.

Pull-down assays of chaperone complexes assembled in insect cells
For the pull down assays presented in Figure 2E, F, Figure 3C, D and Figure S2G and H, chaperone complexes containing $Hsp90_{Sf9}$-Cdc37-CDK4 or $Hsp90_{Sf9}$-Cdc37-CDK6 were generated by co-expressing human GSTCDK4 or GSTCDK6 with human untagged $Cdc37_{(1-348)}$ in *Sf*9 insect cells using the baculovirus expression system as described above. Complexes were purified using the GST tag on the CDK as described in Vaughan *et al*. (Vaughan et al., 2006). Chaperone complexes were separated by SEC (Superdex 200 16/60 (GE Healthcare)). Fractions containing $Hsp90_{Sf9}$-Cdc37-CDK4 or $Hsp90_{Sf9}$-Cdc37-CDK6 were pooled and concentrated. For pull downs, 4.8 µg (0.150 nmol) of biotinylated C-terminally Avi-tagged viral cyclin K (D-type cyclin) or p16INK4a was incubated for 1 hr at 4 ^{o}C with 25 µl NeutrAvidin® Agarose Resin in a toal volume of 60 µl (Thermo Scientific Pierce) in 20 mM Tris pH 7.5, 150 mM NaCl, 5 mM Na_2MoO_4, 0.5 mM EDTA, 0.5 mM TCEP and 0.5% Igepal. A 3-fold molar excess of chaperone complex purified from insect cells was added and incubated for a further hour at 4 ^{o}C. Each pull down was washed as described above and boiled prior to separation by SDS-PAGE. Proteins were visualized by InstantBlue staining.

pRb kinase assay
To assemble the various complexes, 15 µg of Cdc37 was mixed with a molar equivalent of CDK6, and incubated for 1hour at 4 ^{o}C. Vcyclin was then added to appropriate samples at a 1:1 molar ratio CDK6:Vcyclin and incubated for a further hour at 4 ^{o}C. Vcyclin complexes with Cdc37 or CDK6 were generated by mixing proteins at equivalent 1:1 molar ratios. During incubation steps, the samples were manually mixed half-way through. 200 µl of glutathione bead slurry was washed in pRb assay buffer (50 mM HEPES, pH7.5, 10 mM $MgCl_2$, 1 mM DTT, 0.1 mM orthovandate, 1 mM NaF, 0.1 mM EDTA) three times and then 1 mg of GST-pRb encoding pRb residues 379-928 (Kato et al., 1993) was added and incubated for 30 mins at 4 ^{o}C. The beads were pelleted, the excess supernatant removed and then re-suspended to 200 ul volume. 20 µl of GST-pRb–bead slurry was added to each reaction tube, and the volume made up to 100 µl using pRb assay buffer. The kinase reactions were initiated by the addition of ATP to a final concentration of 2 mM, then allowed to proceed for 30 mins at 30 ^{o}C with gentle agitation. The reactions were stopped by addition of 2 x LDS buffer and analysed by SDS-PAGE and subsequent western blotting using an anti-phosphorylated S780 antibody (Abcam, ab173289).

Homogenous time-resolved fluorescence (HTRF)
(a) Direct binding format: In the direct binding assay format glutathione-S-transferase (GST)-tagged cyclin-dependent kinase (CDK) is incubated with biotinylated Avi-tagged protein of interest to form a GSTCDK-protein complex. The complex is then incubated with a Tb-labeled anti-GST antibody and streptavidin-tagged XL665 dye. Formation of a complex brings the Tb and XL665 into proximity so that excitation of the Tb results in emission from the XL665 dye as a result of Förster resonance energy transfer (FRET) between the two probes. To optimize the assay for direct binding measurements the biotinylated protein of interest was initially titrated, over 11 serial dilution points and an additional buffer blank point, against 5 fixed concentrations of either GSTCDK4 or GSTCDK6 (100 nM, 50 nM, 25 nM, 10 nM and 5 nM). Subsequently experiments to assay the various GSTCDK complexes described in Figures 1B; 4C-F; and Figure S2A-D were carried out at a fixed GSTCDK concentration of 10 nM. The concentration of GSTCDK2 and GST used in Figure S1A, B was 25 nM. Concentrations of GSTCDK and the binding protein of interest were prepared in HTRF buffer A (50 mM HEPES, 100 mM NaCl, 1 mM DTT and 0.1 mg/ml BSA) and incubated together for 60 minutes at 4°C or on a shaker at room temperature. 5 nM Tb labeled anti-GST antibody and SAXL665 at 1/8th the concentration of the biotinylated protein, were prepared in HTRF buffer B (50 mM HEPES, 100 mM NaCl and 0.1 mg/ml BSA) and added to each well. The plate was incubated for a further 120 minutes at 4°C, before being scanned. Samples were excited using a wavelength of 337 nm and emission spectra measured at 620 nm and 665 nm (PHERAstar FS (BMG LABTECH)). Binding curves were plotted using GraphPad Prism 6 from which the K_ds were

determined. The curves shown are representative binding curves from at least two runs with each mutant carried out on separate days.

(b) Competition mode: 8 nM GSTCDK4 and 150 nM C-Avi Cdc37, or 6 nM GSTCDK6 and 500 nM C-Avi Cdc37 (the Cdc37 concentration was adjusted to the measured K_d for the CDK-Cdc37 interaction), were added to each well along with a serial dilution of the competitor (viral cyclins, Figure 2A, B; INKs, Figure 3A, B, E, F; CIP/KIPs, Figure 4A, B; ATP competitive inhibitors, Figure 1C, D; or ATP, Figure S1E, F). All dilutions were made using HTRF buffer A (50 mM HEPES, 100 mM NaCl, 1 mM DTT and 0.1 mg/ml BSA) and set up in duplicate. ATP-competitive inhibitor stocks were prepared either in 100% DMSO (LEE011) or water (PD0332991 and LY2835219). LEE011 titrations were carried out at a final DMSO% of 0.1%. The plate was incubated for 60 minutes at 4°C, before 0.5 nM Tb labeled anti-GST antibody and 18.75 nM of SAXL665 (for the CDK4 measurements) or 3 nM Tb labeled anti-GST antibody and 62.5 nM of SAXL665 (for the CDK6 measurements), were added to each well. Concentrations of the Tb antibody and SAXL665 were made up using HTRF buffer B (50 mM HEPES, 100 mM NaCl and 0.1 mg/ml BSA). The plate was incubated for a further 120 minutes at 4°C, before being scanned. Samples were excited using a wavelength of 337 nm and emission spectra measured at 620 nm and 665 nm (PHERAstar FS (BMG LABTECH)). Percentage inhibition graphs were plotted using GraphPad Prism 6, by comparing to a maximum signal, where no competitor is present, and a minimum signal, where no GSTCDK4 or GSTCDK6 is present.

Surface Plasmon Resonance (SPR)
All SPR experiments were performed on a Biacore S200 (GE Healthcare) at 4 °C using SPR buffer (10 mM HEPES pH 7.5, 150 mM NaCl, 3 mM EDTA and 0.05% Tween 20). Samples were centrifuged at 10000 x g for 10 minutes at 4 °C before use. 40 µg/mL GSTCDK4 or 30 µg/mL GSTCDK6 were captured on a surface of goat anti-GST antibody (GE Healthcare) at 5 µL/min for 600s. Antibody capture to the CM5 BIAcore sensor chip was via amine coupling using the standard protocol provided in the GST capture kit (GE Healthcare). Analyte solutions of p16INK4a and p16INK4a mutants at 200 nM, 100 nM, 25 nM, 6.25 nM and 1.56 nM were flowed over the bound GSTCDKs at 30 µL/min for 240s and the dissociation was measured over 600s. Analyte solution of Cdc37 at 4000 nM, 2000 nM, 1000 nM, 250 nM and 62.5 nM were flowed over for 700s and the dissociation measured at 600s. In case of viral cyclins: 500 nM, 250 nM, 62.5 nM, 15.6 nM and 4 nM for 600s and dissociation was measured at 600s. The bound GSTCDKs were removed from the antibody using 10 mM glycine pH 2.1 at 30 µL/min for 240s. The chip was regenerated by capturing fresh GSTCDKs before each analyte concentration run. GST was loaded onto a separate lane at 10 µg/mL for 120s at 5 µL/min to act as a negative control. Sensorgram readings from the GST control were subtracted from GSTCDK sensorgrams to correct for non-specific interactions and bulk effects. K_d values and dissociation rates were calculated using the Biacore S200 Evaluation Software.

Differential Scanning Fluorimetry
A protein/Sypro orange mix containing 5 µM protein and a 1:5,000 dilution of dye in DMSO (as supplied by Sigma Aldrich) was prepared just before plate setup in 10 mM HEPES pH 7.5, 150 mM NaCl, 0.5 mM EDTA and 0.5 mM TCEP. 14.5 µL of the protein/Sypro orange mix was aliquoted into 384-well plates and sealed. Each experiment was done in triplicate. Thermal melting experiments were carried out using an QuantStudio Real Time PCR machine (Applied Biosystems). The plates were first equilibrated at 25°C for 2 min in the PCR machine before starting the thermal melting experiment upon which the plates were heated at 0.05°C per second from 25 to 99°C. The fluorescence intensity was recorded using the ROX filters under continuous collection. Raw fluorescence data were extracted from the QuantStudio Real-Time PCR software and analysed using the Applied Biosystems Protein Thermal Shift software. Calculated derivative Tm values for each of the three repeats were extracted and the average values calculated.

Silver staining
3 µg of complexes GST-CDK4 or GST-CDK6 used in HTRF and SPR assays were run on 10% SDS-PAGE gel (Biorad) for 35 minutes in 1xTGS Buffer (Biorad) at 200 V. Subsequently gels were silver stained using Pierce Silver Stain Kit (ThermoScientific) using manufacturer instructions. The gel was stained until bands started appearing and stopped with 5% acetic acid for 10 minutes, followed by 2x 5 minutes washes with ultrapure water.

Western blotting
*Sf*Hsp90-Cdc37-CDK4 purified from insect cells (10 µg) was incubated with buffer or p16INK4a (20 µg) for 60 minutes on ice and immuno-precipitated with anti-Hsp90 antibodies (abcam AC88 #ab13492) overnight. Bead bound proteins were washed 3x times with Lysis/Wash buffer (ThermoFisher Scientific, #26146). Subsequent to SDS-PAGE samples were western blotted using anti -Hsp90 (Cell Signaling, #4874), -Cdc37 (ThermoFisher Scientific, C1 #MA3-029), -CDK4 (Cell signaling, CDK4 (D9G3E) #12790) and -p16INK4a (Abcam, DCS50.1, #ab16123) antibodies.

Supplemental References

Bieniossek, C., Imasaki, T., Takagi, Y., and Berger, I. (2012). MultiBac: expanding the research toolbox for multiprotein complexes. Trends Biochm Sci *37*, 49-57.

Brown, N.R., Noble, M.E., Lawrie, A.M., Morris, M.C., Tunnah, P., Divita, G., Johnson, L.N., and Endicott, J.A. (1999). Effects of phosphorylation of threonine 160 on cyclin-dependent kinase 2 structure and activity. J Biol Chem *274*, 8746-8756.

Kato, J., Matsushime, H., Hiebert, S.W., Ewen, M.E., and Sherr, C.J. (1993). Direct binding of cyclin D to the retinoblastoma gene product (pRb) and pRb phosphorylation by the cyclin D-dependent kinase CDK4. Genes Dev *7*, 331-342.

Vaughan, C.K., Gohlke, U., Sobott, F., Good, V.M., Ali, M.M., Prodromou, C., Robinson, C.V., Saibil, H.R., and Pearl, L.H. (2006). Structure of an Hsp90-Cdc37-Cdk4 complex. Mol Cell *23*, 697-707.
