# Supplementary material for: Differential Regulation of G1 CDK Complexes by the Hsp90-Cdc37 Chaperone System
Source: Cell Rep. 2017 Oct 31;21(5):1386–98. doi: 10.1016/j.celrep.2017.10.042 (PMC5681435; doi:10.1016/j.celrep.2017.10.042)
Supplement: Document S2. Article plus Supplemental Information [file mmc2.pdf]

Cell Reports

Article

Differential Regulation of G1 CDK Complexes by the Hsp90-Cdc37 Chaperone System

Graphical Abstract

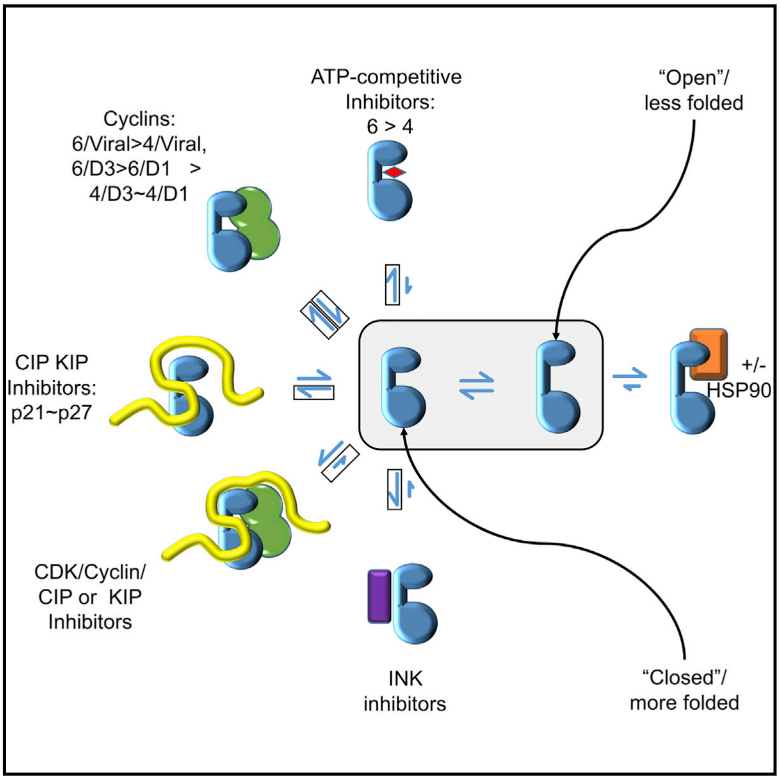

Authors

Stephen T. Hallett, Martyna W. Pastok, R. Marc L. Morgan, ..., Martin E.M. Noble, Laurence H. Pearl, Jane A. Endicott

Correspondence

laurence.pearl@sussex.ac.uk (L.H.P.), jane.endicott@ncl.ac.uk (J.A.E.)

In Brief

Hallett et al. reconstitute CDK4/6 client kinase handover from Cdc37-Hsp90 to CDK regulatory partners and propose a model for the assembly factor activity of CIP/KIP CDK inhibitors. They find that CDK4/6 inhibitors in clinical use can displace G1 CDKs from the Cdc37-Hsp90 chaperone system at submicromolar concentrations.

Highlights

- CDK inhibitors and D-type cyclins competitively sequester CDK4/6 from Cdc37-Hsp90
- Binding of ATP-competitive inhibitors causes CDK4/6 to be displaced from Cdc37-Hsp90
- Cancer-associated p16INK4a mutants distinguish CDK4 and CDK6
- A model for the assembly factor activity of CIP/KIP CDK inhibitors is proposed

Hallett et al., 2017, Cell Reports *21*, 1386–1398
October 31, 2017 © 2017 The Author(s).
https://doi.org/10.1016/j.celrep.2017.10.042

CellPress

OPEN ACCESS
CellPress

Cell Reports
Article

Differential Regulation of G1 CDK Complexes by the Hsp90-Cdc37 Chaperone System

Stephen T. Hallett,[1,4] Martyna W. Pastok,[1,4] R. Marc L. Morgan,[2,3,4] Anita Wittner,[1] Katie L.I.M. Blundell,[2] Ildiko Felletar,[2] Stephen R. Wedge,[1] Chrisostomos Prodromou,[2] Martin E.M. Noble,[1] Laurence H. Pearl,[2,*] and Jane A. Endicott[1,5,*]
[1]Newcastle Cancer Centre, Northern Institute for Cancer Research, Paul O'Gorman Building, Medical School, Newcastle University, Framlington Place, Newcastle upon Tyne NE2 4HH, UK
[2]Genome Damage and Stability Centre, School of Life Sciences, University of Sussex, Science Park Road, Falmer, Brighton BN1 9RQ, UK
[3]Present address: Protein Crystallography Facility, Centre for Structural Biology, Flowers Building, Department of Life Sciences, Imperial College London, London SW7 2AZ, UK
[4]These authors contributed equally
[5]Lead Contact
*Correspondence: laurence.pearl@sussex.ac.uk (L.H.P.), jane.endicott@ncl.ac.uk (J.A.E.)
https://doi.org/10.1016/j.celrep.2017.10.042

SUMMARY

Selective recruitment of protein kinases to the Hsp90 system is mediated by the adaptor co-chaperone Cdc37. We show that assembly of CDK4 and CDK6 into protein complexes is differentially regulated by the Cdc37-Hsp90 system. Like other Hsp90 kinase clients, binding of CDK4/6 to Cdc37 is blocked by ATP-competitive inhibitors. Cdc37-Hsp90 relinquishes CDK6 to D3- and virus-type cyclins and to INK family CDK inhibitors, whereas CDK4 is relinquished to INKs but less readily to cyclins. p21CIP1 and p27KIP1 CDK inhibitors are less potent than the INKs at displacing CDK4 and CDK6 from Cdc37. However, they cooperate with the D-type cyclins to generate CDK4/6-containing ternary complexes that are resistant to cyclin D displacement by Cdc37, suggesting a molecular mechanism to explain the assembly factor activity ascribed to CIP/KIP family members. Overall, our data reveal multiple mechanisms whereby the Hsp90 system may control formation of CDK4- and CDK6-cyclin complexes under different cellular conditions.

INTRODUCTION

A large proportion of the human kinome requires chaperoning through the Hsp90 pathway to be activated and/or assembled into functional complexes. Protein kinases are recognized and bound by Cdc37, which acts as a gatekeeper to direct this particular class of client proteins to Hsp90 (Prodromou and Pearl, 2014). Molecular details of how Cdc37 recognizes diverse protein kinase sequences have been elucidated recently by determination of a structure of an Hsp90-Cdc37-CDK4 complex by cryoelectron microscopy (Verba et al., 2016). This structure confirms earlier studies that implicated sequence features of the protein kinase N-terminal lobe and ATP-binding cleft as contributing to the Cdc37 binding site (Citri et al., 2006; Eckl et al., 2015; Polier et al., 2013; Xu et al., 2005; Zhao et al., 2004). Cdc37-Hsp90 associates with client kinases both during their initial folding and after they have reached their mature state. It has been hypothesized that the strength of this interaction is dictated in large part by differences in the thermal and conformational stabilities of the client protein (Taipale et al., 2012). From insights provided by the Hsp90-Cdc37-CDK4 structure, it has been hypothesized that these differences in stability manifest themselves as a differential propensity of kinase domains to adopt an open state in which the N- and C-terminal lobes are separated and can make alternative interactions with Cdc37 and Hsp90 (Verba et al., 2016). Indeed, Cdc37 has been proposed to sort client kinases by testing their ability to resist local unfolding (Keramisanou et al., 2016). Although weak client kinases can be hypothesized to be proteins that readily escape Cdc37 because they have an elevated stability, strong clients remain associated with Cdc37-Hsp90 because they are metastable. As a result, they are held in a protected state until relinquished to an appropriate partner. Such a model would explain the observed ability of the Cdc37-Hsp90 complex, through Cdc37, to both recognize diverse protein kinase sequences with widely different affinities and to sequester them until an appropriate binding partner is available (Boczek et al., 2015; Keramisanou et al., 2016; Verba et al., 2016).

Members of the cyclin-dependent protein kinase (CDK) family differ substantially in their dependency on the Cdc37-Hsp90 pathway (Taipale et al., 2012). CDKs that regulate the cell cycle all require cyclin binding for full activity (Morgan, 2007). Given the expression profiles of their cognate cyclins, the cell cycle CDKs, CDKs 1, 2, 4, and 6, may exist during some stages of the cell cycle in a non-cyclin-bound inactive state. Indeed, early studies to purify CDK complexes identified monomeric CDK1 and CDK2 in cell extracts (Arooz et al., 2000; Gu et al., 1993). In contrast, many CDK4 partner proteins function in pathways that regulate protein folding and complex assembly, suggesting that CDK4 may not be stable as a monomer (Jirawatnotai et al., 2014). Consistent with these observations, CDK1 and CDK2 have no dependency on Cdc37-Hsp90, whereas CDK4 and CDK6 are acknowledged Cdc37-Hsp90 client proteins (Lamphere et al., 1997; Parry et al., 1999; Smith et al., 2015; Stepanova et al., 1996; Taipale et al., 2012). These observations suggest

This is an open access article under the CC BY license (http://creativecommons.org/licenses/by/4.0/).

that monomeric CDK1 and CDK2 are relatively stable in the absence of their cognate binding partners but that CDK4 and CDK6 distribute into larger complexes that can include Cdc37 and Hsp90.

Cell cycle CDK-cyclin complexes are regulated by association with members of two families of CDK inhibitors (CKIs) (Hunter and Pines, 1994). The CIP/KIP family binds to both the cyclin and CDK subunits to inhibit CDKs 1, 2, 4, and 6 (Russo et al., 1996), whereas members of the INK family specifically bind to monomeric CDK4 and CDK6 (Brotherton et al., 1998; Russo et al., 1998). Although the INK and cyclin D binding sites on CDKs do not overlap, INK binding alters the relative disposition of the N- and C-terminal lobes of the CDK4 or CDK6 kinase domain and, thus, through allostery, weakens the CDK interaction with its cyclin partner (Brotherton et al., 1998; Russo et al., 1998). In apparent contradiction to their role as CDK inhibitors, members of the CIP/KIP family have also been described as assembly factors that promote the formation of CDK4/6-cyclin D complexes during G1 ((Bisteau et al., 2013; Blain et al., 1997; Cheng et al., 1999; LaBaer et al., 1997; Soos et al., 1996; Zhang et al., 1994; reviewed in Sherr and Roberts, 1999), and, dependent on the state of CKI phosphorylation, ternary CDK-cyclin-CKI complexes can be catalytically active (Larrea et al., 2008; Ray et al., 2009). This activity is also proposed to promote G1 progression by sequestering CIP/KIP CKIs to create an environment in which the activity of CDK2 can rise, driven by increasing cyclin E expression (Sherr et al., 2016). Taken together, previous studies suggest that the relative importance of these apparently contradictory CKI roles in regulating cell cycle progression are dependent on cell type and prevailing conditions.

The molecular details of how any client kinase is relinquished by Cdc37-Hsp90 have not been elaborated. Cdc37-Hsp90 association with CDK4 *in vivo* is mutually exclusive with either cyclin (Stepanova et al., 1996) or p16INK4a (Lamphere et al., 1997) binding, suggesting that either protein might be a suitable partner to which Cdc37-Hsp90 would transfer its client. In this study, we set out to characterize the interactions of CDK4 and CDK6 with the Cdc37-Hsp90 chaperone pathway and to determine whether known CDK binding proteins can displace CDK4 or CDK6 from Cdc37-Hsp90 complexes. We demonstrate in cell-free assays that CDK4 and CDK6 can both interact with Cdc37 and Cdc37-Hsp90 but differ considerably in their affinities. CDK6 is a relatively weak client and can readily be displaced from Cdc37 by members of the INK family or D-type cyclins. CDK4, in contrast, is a strong client and binds tightly to Cdc37 and to Cdc37-Hsp90. We show that Cdc37-Hsp90 will relinquish CDK4 to members of the INK family but not to D-type cyclins. We find that cancer-associated p16INK4a mutations differ in their modes of action toward CDK4 and CDK6 and in their abilities to displace CDK4 and CDK6 from Cdc37. The CKIs p21CIP1 and p27KIP1 cooperate with the D-type cyclins to generate CDK4/6-containing ternary complexes that are resistant to cyclin D displacement by Cdc37, suggesting a molecular mechanism for CIP/KIP assembly factor activity. Our results demonstrate that CDK4 and CDK6 are distinguished as clients of the Cdc37/Hsp90 system by cyclin and INK partners.

RESULTS

Monomeric CDKs Exhibit Differing Affinities for Cdc37

To evaluate whether the pattern of dependency on Cdc37-Hsp90 that is observed in cells can be recapitulated with purified proteins, CDKs 2, 4, and 6 were tested for their ability to bind to Cdc37 *in vitro*. Using a homogeneous time-resolved fluorescence (HTRF) assay (Figure 1A), the measured affinities of CDK4 and CDK6 for Cdc37 were approximately 90 nM and greater than 500 nM respectively, whereas no interaction could be detected between Cdc37 and CDK2 (Figure 1B; Table 1; Figure S1). The interactions were also tested using surface plasmon resonance (SPR) immobilizing GSTCDK4 or GSTCDK6, and similar affinities were measured (Figures S1C and 1D). These affinities are comparable with the value of approximately 200 nM reported for the Cdc37-B-Raf^{V600E} kinase domain interaction, measured by isothermal titration calorimetry (Polier et al., 2013), and reinforce the idea that stable interaction with Cdc37 is the primary determinant of whether a kinase is an Hsp90 client. As previously reported, CDK4 and CDK6 can be distinguished by their affinities for Cdc37 (Stepanova et al., 1996; Taipale et al., 2012). We next set out to determine how known CDK4/6 regulators might affect the interactions between CDK4 and CDK6 with Cdc37.

ATP-Competitive Inhibitors Antagonize the Interaction between CDK4 or CDK6 and Cdc37

A general model has been proposed for the action of protein kinase ATP-competitive inhibitors as agents that antagonize the Cdc37-client protein kinase interaction, depriving the client of access to the Hsp90 chaperone system and, thereby, promoting its ubiquitylation and proteasomal degradation (Polier et al., 2013). To test whether this model extends to CDK4 and CDK6, we adapted the HTRF assay to run in a competition format. ATP and three potent CDK4/6 inhibitors, PD0332991 (palbociclib), LEE011, (ribociclib), and LY2835219 (abemaciclib), with measured half-maximal inhibitory concentration (IC_{50}) values against CDK4/6-cyclin D of 11/15 nM, 10/39 nM, and 2/5 nM, respectively (values compiled in Sherr et al., 2016) were titrated into pre-formed CDK4- or CDK6-Cdc37 complexes (Figures 1C and 1D). All three inhibitors were able to displace Cdc37 from CDK partners. PD0332991 is more potent than LEE011 at displacing CDK4 and CDK6 from Cdc37, and against CDK6, LY2835219 is as effective as PD0332991. CDK6 was more readily displaced from Cdc37 than CDK4 by PD0332991 and LEE011. ATP was unable to displace Cdc37 at physiological (millimolar) concentrations (Figures S1E and S1F). Despite repeated attempts, we were unable to derive reproducible inhibition curves for the activity of LY2835219 toward CDK4-Cdc37. This result suggests that the binding of LY2835219 to CDK4 is not described by a straightforward reversible 1:1 interaction under our assay conditions or may interfere with the assay. Taken together, these results support a model in which Cdc37 and ATP-competitive inhibitor binding to CDK4 or CDK6 are mutually exclusive and suggest that CDK4/6-selective inhibitors also exert at least part of their therapeutic effects through depriving CDK4/6 of access to the Cdc37-Hsp90 system.

OPEN ACCESS
CellPress

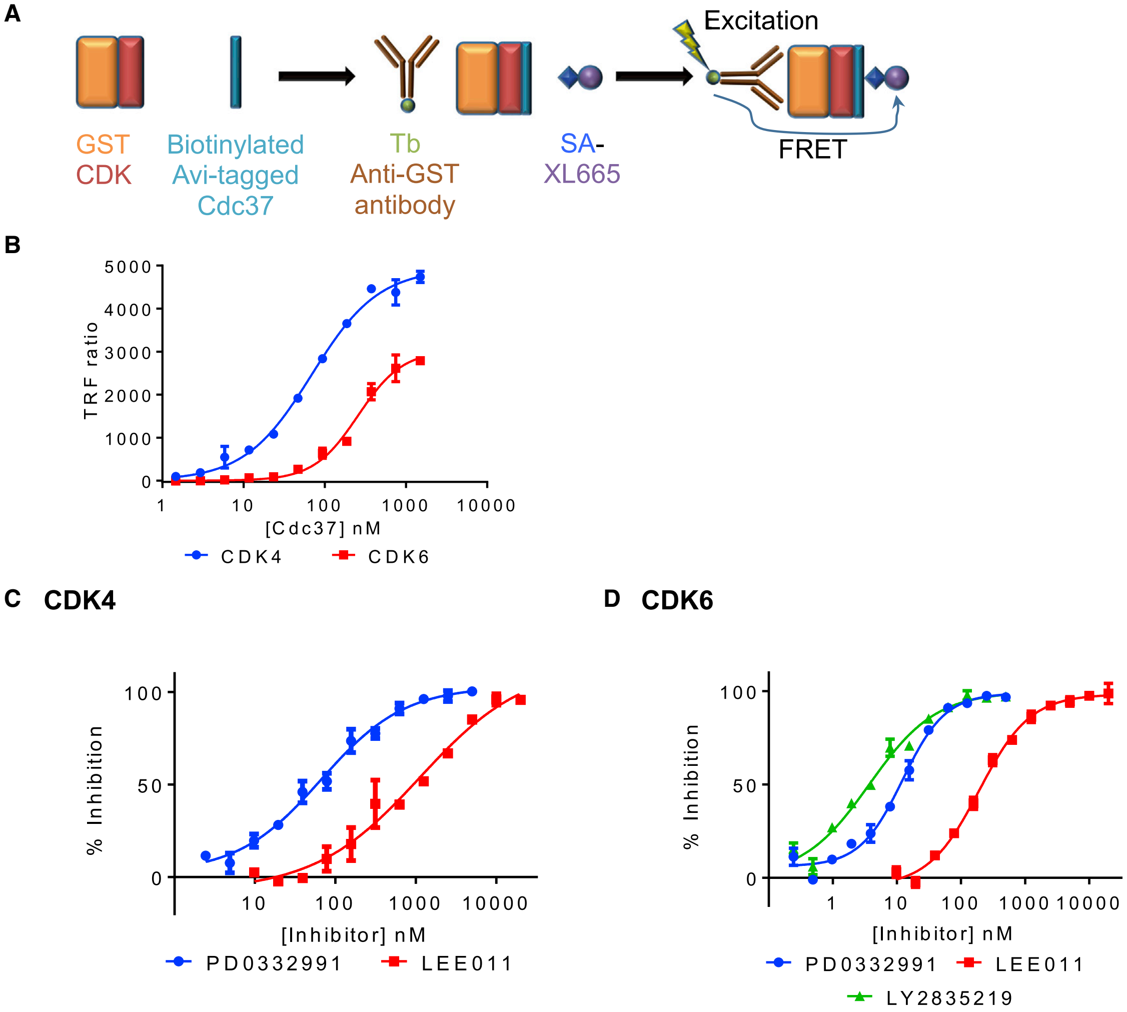

Figure 1. CDK4 and CDK6 Bind to Cdc37

(A) Homogeneous time-resolved fluorescence (HTRF) assay format.

(B) Cdc37 binding to CDK4 (blue line) and to CDK6 (red line) measured by HTRF. The concentration of CDK4 and CDK6 used in these assays was 10 nM. From SPR titrations between GSTCDK4 or GSTCDK6 and Cdc37, it can be calculated that 34% and 20%, respectively, of the CDK4 and CDK6 are active (Figures S1C and S1D).

(C and D) ATP-competitive inhibitors can displace Cdc37 from a CDK4-Cdc37 (C) or CDK6-Cdc37 (D) complex. CDK4 and CDK6 concentrations were 8 nM and 6 nM, respectively. HTRF measurements were carried out in duplicate and repeated on 3 separate days. The error bars indicate SD.

See also Figure S1.

Cyclins Distinguish CDKs as Clients of the Cdc37/Hsp90 System

Cdc37 and cyclin D binding to CDK4 is mutually exclusive *in cellulo* (Lamphere et al., 1997; Stepanova et al., 1996), suggesting that D-type cyclins could be suitable partners to which the Cdc37-Hsp90 complex would hand over its client CDK. Unfortunately, recombinant monomeric cyclin D is unstable and prone to aggregation, so we were first obliged to use viral D-type cyclins from Herpesvirus saimiri and Kaposi's sarcoma-associated herpesvirus (referred to as Vcyclin and Kcyclin, respectively) as surrogates. These viral cyclins bind to CDK4 and CDK6 to promote their activity through G1 following viral infection (Li et al., 1997; Swanton et al., 1997). The crystal structure of CDK6-Vcyclin shows that cyclin engagement activates the CDK6 to form a heterodimer whose overall organization is reminiscent of activated CDK2-cyclin A (Schulze-Gahmen and Kim, 2002). However, the viral cyclin is distinguished from cyclin D by the absence of a cyclin recruitment site that binds to the RXL recruitment motif that assists binding of various substrates and CIP/KIP inhibitors (Schulze-Gahmen and Kim, 2002; Swanton et al., 1997).

Using HTRF (Figures S2A and S2D) and SPR (Figures S2E and S2F), both viral cyclins bind to CDK4 and to CDK6, albeit with a slightly lower affinity for CDK4 (Table 1). To test whether the viral cyclins can displace Cdc37 from a CDK-Cdc37 complex, glutathione S-transferase (GST)-tagged CDK4 or CDK6 was first incubated with biotinylated C-terminally Avi-tagged Cdc37 and then

Table 1. Dissociation Constants for the Binding of Cdc37 and Cyclins to CDK4 or CDK6

	CDK4	CDK6
Cdc37	92 ± 29	460 ± 230[a]
Vcyclin	151 ± 11	14.9 ± 8.8
Kcyclin	24.9 ± 9.1	10.0 ± 3.5

The values (nanomolar) are derived from homogeneous time-resolved fluorescence measurements. Errors represent the SD from the mean. See also Figure S2.

[a]Analyzed values are close to the signal-to-noise limited sensitivity of the assay.

titrated against increasing concentrations of unlabeled Vcyclin or Kcyclin. Both viral cyclins were only just able to completely dissociate a complex of CDK4-Cdc37 at the highest concentration assayed (1 μM; Figure 2A) but could relatively readily displace CDK6 from a CDK6-Cdc37 complex (100% inhibition achieved at concentrations around 100 nM; Figure 2B). Our results demonstrate that the viral cyclins can distinguish Cdc37-CDK4 and Cdc37-CDK6 complexes and confirm that Cdc37 and cyclin binding to CDK4/6 is mutually exclusive.

We next tested whether, as would be predicted by the above results and an equilibrium binding model, authentic D-type cyclins relinquish CDK4 but not CDK6 when challenged with increasing concentrations of Cdc37. FLAG-tagged Cdc37 was bound to beads, incubated with either CDK4-cyclin D1 or D3 or CDK6-cyclin D1 or D3, and then the bound fractions were analyzed by SDS-PAGE (Figure 2). Under these conditions, CDK4 binds to the Cdc37 beads, and cyclin D1 and cyclin D3 cannot be detected in the pull-down complexes (Figure 2C). This result suggests that the CDK4-cyclin D1/D3 complexes undergo substantial reversible disassociation, allowing the CDK4 to redistribute into a complex with Cdc37. As would be predicted from the behavior of CDK6 toward the viral cyclins, the CDK6-cyclin D3 complex was stable, and CDK6 binding to Cdc37 was not detectable (Figure 2D). However, the CDK6-cyclin D1 complex showed a different pattern of activity: it resembles CDK4-cyclin D1/D3 and undergoes detectable dissociation, resulting in the formation of a CDK6-Cdc37 complex. Taken together, these results show that cyclin D1 and cyclin D3 can be distinguished by their respective activities in dissociating Cdc37-CDK4 and Cdc37-CDK6 complexes.

We next sought to determine to what extent the presence of Hsp90β affects the ability of a cyclin partner to disrupt the association of CDK4 and CDK6 with Cdc37. We assembled complexes of CDK4 and CDK6 with Cdc37-Hsp90β in cells by transfecting *Spodoptera frugiperda* (insect) cells with GSTCDK4 or GSTCDK6 and untagged $Cdc37_{(1–348)}$. This strategy permits the co-purification of GSTCDK-Cdc37 and GSTCDK-Cdc37-Hsp90 complexes that incorporate the insect cell Hsp90 in stoichiometric amounts (Vaughan et al., 2006). The high degree of sequence similarity (>70%) between human and *S. frugiperda* Hsp90 (SfHsp90) supports a model in which the insect protein makes authentic interactions with the human CDK and Cdc37 proteins. Both binary and ternary complexes were purified by first exploiting the GST tag and then by a subsequent size-exclusion chromatography step (Figures 2E and 2F, input lanes). These GSTCDK-Cdc37 and GSTCDK-Cdc37-SfHsp90 complexes were then characterized in a pull-down assay using Avi-tagged D-type cyclins as the bait protein. The patterns of CDK4 and CDK6 behavior bound to Cdc37 or to Cdc37-SfHsp90 are similar in this pull-down assay. Kcyclin could extract CDK6 from CDK6-Cdc37 and CDK6-Cdc37-SfHsp90 (Figure 2F). The binary and ternary CDK4 complexes were refractory to disruption (Figure 2E). To confirm that the CDK6-Kcyclin complex that was generated following viral cyclin capture of the CDK6 was authentic, its kinase activity was tested using retinoblastoma protein (Rb) as a substrate (Figure S2I). CDK6-Kcyclin displayed robust activity toward Rb, as measured by phosphorylation of S780.

Taken together, these results demonstrate that cyclins can distinguish CDKs as clients of the Cdc37/Hsp90 system. They suggest that cyclin D1 and cyclin D3 have more comparable affinities for CDK4 than CDK6 and that cyclin D3 binds more tightly to CDK6 than cyclin D1. The ability of cyclins to displace their cognate CDKs from Cdc37 can be understood in terms of the relative affinities of each CDK for Cdc37 and cyclin partners. This model predicts that, in a cellular context, the efficiency of CDK4 and CDK6 displacement from Cdc37 will depend on the identity and concentration of the competing cyclin.

CKIs Also Distinguish CDKs as Clients of the Cdc37/Hsp90 System

Members of the CIP/KIP and INK families of CKIs also bind to CDKs and might also be partners to which the Cdc37 would hand over the CDK. Using the HTRF assay, we determined that CDK4 and CDK6 bind tightly to p16INK4a, with K_d values in the low nanomolar range. However, because these dissociation constants correspond to less than the concentration of bait (GSTCDK) used in the HTRF assay, they represent upper estimates for the affinity of each interaction. To determine more accurate K_d values, we re-evaluated the interactions by SPR (Figures S3A and S3B; Table 2). In these experiments, GSTCDK4 or GSTCDK6 was captured on the chip, and untagged p16INK4a was flowed over as the ligand. The accuracy of the K_d values determined by fitting the kinetic curves (k_{on}, k_{off}) are compromised by the accuracy with which k_{off} can be determined. However, they do indicate very tight association with calculated K_d values of 0.87 ± 0.42 nM and 0.26 ± 0.16, respectively, for the CDK4- and CDK6-p16INK4a interactions.

We then assayed the abilities of the four authentic INK proteins to displace Cdc37 from CDK4- or CDK6-Cdc37 complexes. Addition of increasing concentrations of each authentic INK to CDK4-Cdc37 (Figure 3A) or CDK6-Cdc37 (Figure 3B) resulted in a loss of fluorescent signal, demonstrating that INK and Cdc37 binding to CDK4 and CDK6 is mutually exclusive. In this assay, the determination of the K_d values is again limited by the component concentrations so that, in each case, the ability of the authentic INK to displace Cdc37 from a CDK-Cdc37 complex can be given an upper value of approximately 8 or 6 nM, respectively (which is the concentration of the GSTCDK4 or GSTCDK6 component used in the assay). To confirm the activity of p16INK4a toward the CDK4/6-Cdc37 interaction in the context of a complex with Hsp90, the GSTCDK-Cdc37 and

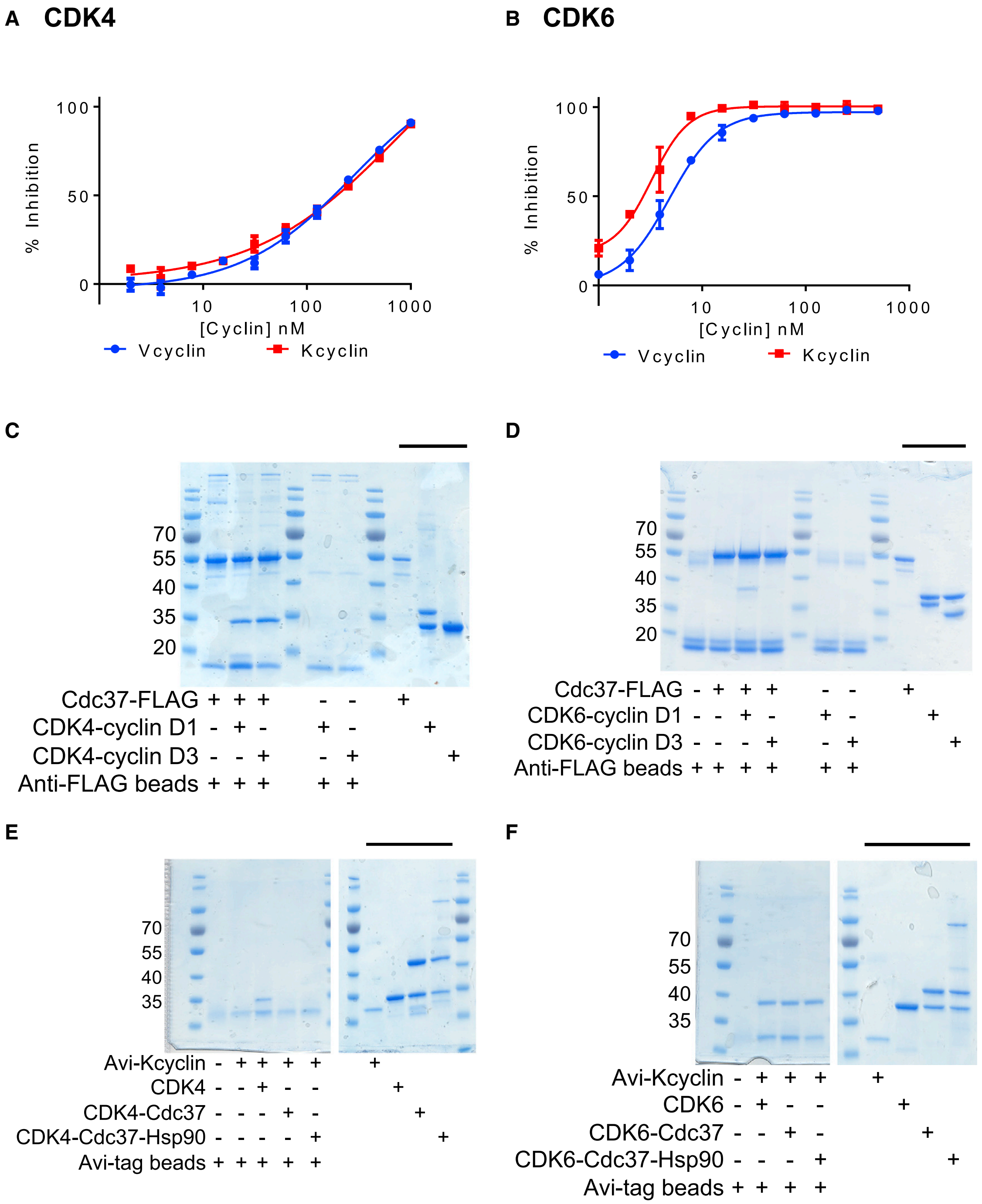

Figure 2. Cyclin and Cdc37 Binding to CDKs Are Mutually Exclusive

(A and B) Kcyclin (red) and Vcyclin (blue) readily displace Cdc37 from CDK6 (B) but not from CDK4 (A). The concentrations of CDK4- and CDK6-containing cyclin D complexes used in the assays were 8 nM (CDK4) and 6 nM (CDK6), respectively. HTRF measurements were carried out in duplicate and repeated on 3 separate days. The error bars indicate SD.

(C and D) FLAG-tagged Cdc37 was co-incubated with CDK4-cyclin D1/D3 (C) or CDK6-cyclin D1/D3 (D) and then analyzed by SDS-PAGE and subsequent InstantBlue staining. The upper band present in the CDK6-cyclin samples is CDK6, whereas the lower band is the cyclin D. The upper band of the CDK4-cyclin D1 complex is cyclin D1, whereas the lower band is CDK4. CDK4 and cyclin D3 have very similar masses and, when analyzed together, are not resolved. Inputs are marked by the black bars above the right hand side lanes. CDK4, but not cyclin D1 or D3, is detected bound to the FLAG-tagged Cdc37 beads (C), indicating that Cdc37 has displaced CDK4 from either cyclin partner. In contrast, CDK6 is not detectable bound to Cdc37 when bound to cyclin D3 but is displaced from cyclin D1 following incubation with Cdc37 (D).

(legend continued on next page)

Table 2. Characterization of p16INK4a and p16INK4a Mutants

	CDK4 (K_d, nM)	CDK6 (K_d, nM)	T_m (°C)	ΔT_m (versus p16INK4a) (°C)
p16INK4a	0.87 ± 0.42	0.26 ± 0.16	47.5 (±1.9)	0.0
p16D84N	ND	59.7 ± 12.0	45.1 (±1.9)	−1.8 (±0.10)
p16D108N	2.56 ± 0.49	0.28 ± 0.10	36.4 (±3.1)	−10.5 (±1.29)
p16M53I	ND	0.73 ± 0.15	42.7 (±0.47)	−4.9 (±2.33)
p16M53E	ND	1.56 ± 0.19	43.2 (±0.38)	−4.6 (±1.66)

Dissociation constants for the binding of wild-type p16INK4a and each mutant to CDK4 or CDK6 are derived from SPR measurements. The T_m for wild-type p16INK4a and each mutant was determined by DSF. Errors represent the SD from the mean. ND, not determined. See also Figure S3.

GSTCDK-Cdc37-SfHsp90 complexes purified from insect cells were characterized in a pull-down assay using Avi-tagged p16INK4a as the bait protein. Confirming the earlier results, Avi-tagged p16INK4a was able to displace both CDK4 and CDK6 from complexes containing Cdc37 and Cdc37-SfHsp90 (Figures 3C and 3D).

Mutant forms of p16INK4a are associated with cancer susceptibility (see http://www.tumorportal.org) and would be predicted to have reduced affinity for CDK4 or CDK6. We reviewed the missense *CDKN2A* sequences compiled at http://www.tumorportal.org and selected the following p16INK4a residues for further study: M53, D84, and D108. These residues are frequently mutated, and, as inferred by inspection of the crystal structure of CDK6-p16INK4a, they would be predicted to interfere with the p16INK4a-CDK interaction (Brotherton et al., 1998). Indeed, p16D84N, in which D84 is mutated to an asparagine (Ruas et al., 1999), and p16M53I, in which M53 is mutated to an isoleucine (Harland et al., 1997), have been reported to show reduced binding to CDK4 and to CDK6.

We first confirmed this activity for p16D84N. SPR allowed a direct quantitative comparison and highlights the rapid dissociation rate that is an acquired characteristic of this mutant, leading to a significant reduction in its binding to both CDK4 (binding could be detected but not quantified) and CDK6 (K_d, 60 nM) (Figures S3C and S3D; Table 2). Loss of this aspartate side chain removes a key hydrogen bond to R31 of CDK6 or R24 of CDK4, a residue that is also mutated in several cancer types (Smith-Sørensen and Hovig, 1996; http://www.tumorportal.org).

Differences in the affinity of p16M53I toward CDK4 and CDK6 are also apparent (Figures S3E and S3F; Table 2). Binding of the p16M53I mutant to CDK4 and CDK6, measured by SPR, had k_{on} values comparable with those of wild-type p16INK4a, whereas the dissociation rates of the mutant protein complexes differed markedly from those of wild-type p16 and depended also on the identity of the CDK. Compared with wild-type p16INK4a (Figure S3B), p16M53I showed an approximately 10-fold higher rate of dissociation from CDK6 (Figure S3F), and its dissociation from CDK4 was substantially increased (Figure S3E). Taking the SPR results together, the p16M53I mutant has a similar affinity for CDK6 (K_d 0.73 ± 0.15 nM) as p16INK4a (K_d 0.26 ± 0.16 nM) but a much reduced affinity for CDK4 (binding could be detected but not quantified; Table 2). A p16M53E mutant was also assayed by SPR (Figures S3G and S3H; Table 2) and showed equivalent behavior. Methionine 53 lies at the INK-CDK6 interface, where its side chain points into the CDK6 active site toward a conserved aspartate (CDK6 D104, CDK4 D99) that sits below the ribose hydroxyls of ATP (PDB: 1BI7, Russo et al., 1998; 1BI8, Brotherton et al., 1998).

In contrast, the activity of p16D108N (D108 mutated to an asparagine) was very similar to that of p16INK4a (Figures S3I and S3J; Table 2). Based on these results, we hypothesized that reduced protein stability might mediate this mutant's effects. To test this hypothesis, we used differential scanning fluorimetry (DSF) to determine the melting temperature ™ values for each of the p16INK4a species (Table 2). Authentic p16INK4a is a stable protein (T_m 47.5°C ± 1.9°C), and the introduction of mutations M53I and D84N resulted in small destabilizations (T_m values of 42.7°C ± 0.47°C and 45.1°C ± 1.9°C, respectively). However, DSF reveals that the p16D108N mutant is distinguished by its significantly depressed melting temperature (T_m value of 36.4°C ± 3.1°C) relative to p16INK4a. This result suggests that this mutation destabilizes p16INK4a, leading to reduced levels of expression rather than inhibiting the interaction with CDK4 or CDK6.

Presuming an equilibrium binding model, a hypothesis that can be proposed from our results is that p16INK4a mutants will differ in their abilities to displace Cdc37 from CDK4 and CDK6. To test this hypothesis, we assayed the p16INK4a mutants in the HTRF displacement assay (Figures 3E and 3F). p16D84N and p16D108N behaved as expected: p16D84N was unable to displace CDK4 or CDK6 from Cdc37 (Figures 3E and 3F, gray curves), whereas p16D108N was as effective as wild-type p16INK4a in displacing CDK6 (Figure 3F, blue curve) and showed only a modest reduction in activity toward CDK4 (Figure 3E, blue curve). In agreement with our hypothesis, p16M53I is as effective as authentic p16INK4a at displacing Cdc37 from CDK6 (Figure 3F, cyan curve) but is ineffective at displacing Cdc37 from CDK4 (Figure 3E, cyan curve).

(E and F) Kcyclin binds to CDK4 (E) and CDK6 (F) and is able to displace CDK6 (F) but not CDK4 (E) from CDK-Cdc37 and CDK-Cdc37-Hsp90β complexes assembled in insect cells. The uncropped gels that include the control lanes to confirm that CDK4, CDK6, Cdc37, and Hsp90 do not stick non-specifically to Avi-tagged beads are included as Figures S2G and S2H. Inputs are marked by the black bars above the right hand side lanes. Samples were analyzed by SDS-PAGE and subsequent InstantBlue staining.
See also Figure S2.

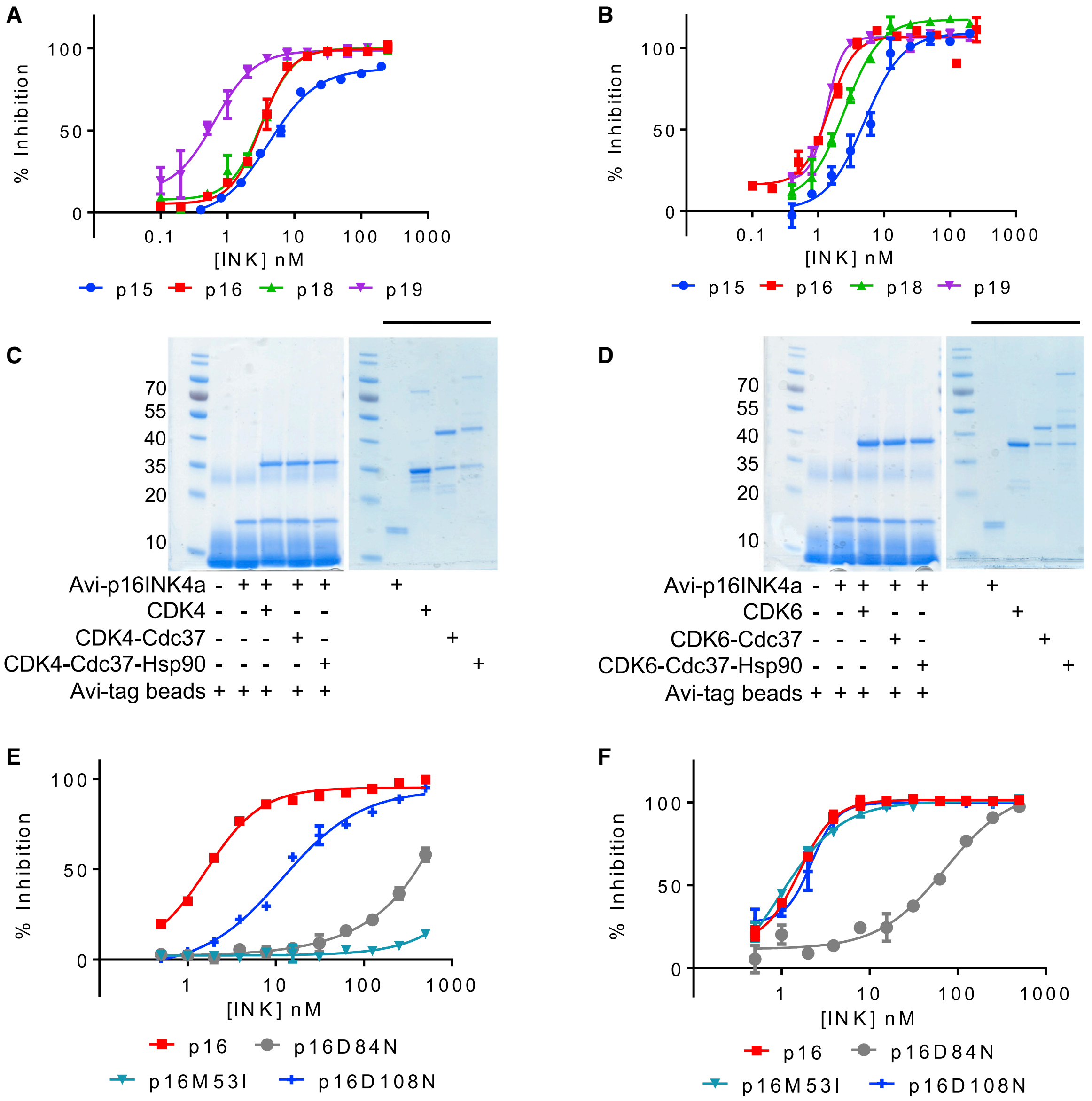

Figure 3. CKI-Mediated Displacement of Cdc37 from CDK4-Cdc37 and CDK6-Cdc37 Complexes

(A and B) Displacement of Cdc37 from CDK4 (A) or CDK6 (B) by members of the INK family.

(C and D) p16INK4a is able to displace CDK4 (C) and CDK6 (D) from CDK-Cdc37 and CDK-Cdc37-Hsp90β complexes assembled in insect cells. The uncropped gels that include the control lanes to confirm that CDK4, CDK6, Cdc37, and Hsp90 do not stick non-specifically to Avi-tagged beads are included as Figure S4.

(E and F) Mutant p16INK4a varies in its ability to displace Cdc37 from a CDK4-Cdc37 complex (E) or a CDK6-Cdc37 complex (F). The concentrations of CDK4 and CDK6 were 8 nM and 6 nM, respectively.

The HTRF measurements presented in (A) and (B) were carried out in duplicate and repeated on 3 separate days. The measurements presented in (C) and (D) were carried out in duplicate and independently repeated twice. The error bars indicate SD. See also Figures S3 and S4.

Taken together, these results suggest that not all p16INK4a mutations detected in tumor cells act through the same mechanism. The observation that p16M53I is as effective at binding to CDK6 as the authentic p16INK4a sequence suggests that this mutation may only be effective in settings where cell cycle progression is driven by CDK4. Furthermore, it can be hypothesized that clinically significant changes to the p16INK4a sequence might not only affect the regulation of CDK4 and CDK6 activity by affecting the direct binding of p16INK4a but also by affecting the ability of p16INK4a to inhibit the interactions of CDK4 and CDK6 with their regulators.

CDK-Cyclin D Complexes Selectively Resist Disruption by Cdc37 in the Presence of p21CIP1 or p27KIP1

Members of the CIP/KIP family have been proposed to act as assembly factors for CDK4/6-cyclin D complexes during G1.

As demonstrated above, D-type cyclin alone is unable to displace Cdc37 from CDK4, and CDK4 bound to cyclin D1 or D3 or CDK6 bound to cyclin D1 redistribute into complexes containing Cdc37. From these results, we hypothesized that addition of p21CIP1 or p27KIP1 might stabilize CDK4/6-cyclin D complexes relative to Cdc37 or Cdc37-Hsp90 complexes with CDK4 or CDK6.

Using the HTRF assay, we first observed that full-length p27KIP1 (p27FL) displaced Cdc37 from CDK4-Cdc37 or CDK6-Cdc37, albeit significantly less efficiently than the INKs (Figures 4A and 4B). The crystal structure of a CDK2-cyclin A-p27KIP1 complex reveals that the N-terminal sequence of p27KIP1 has a significant interaction with the CDK N-terminal lobe that might explain this activity (Russo et al., 1996). Accordingly, constructs related to the co-crystallized fragment of p27KIP1 (residues 1–106, termed p27M) and to that part of the construct that was resolved in the electron density of that complex (p27KIP1 residues 23–106, termed p27S) were assayed for their ability to displace Cdc37 from CDKs 4 and 6. We also assayed corresponding fragments of p21CIP1 (p21M/S, residues 1/9–87). Each of these constructs retains the ability to displace Cdc37 (Figures 4A and 4B), suggesting that Cdc37 displacement activity resides in residues contained within the "S" fragment constructs of CIP/KIP proteins. However, p27FL was the most potent p27KIP1 construct tested and showed equivalent activity toward CDK4 and CDK6, suggesting that additional p27KIP1 residues C-terminal to residue 106 may contribute to Cdc37 displacement.

To find out whether the CIP/KIP CKIs can cooperate with cyclin D isoforms to generate a stable ternary CDK4 complex that is resistant to displacement by Cdc37, we next purified GSTCDK4-cyclin D1 and D3 and GSTCDK6-cyclin D1 and D3 complexes assembled in insect cells, incubated them with excess p21M or p27M, and isolated the complexes by size-exclusion chromatography. We then used the HTRF assay to measure the association of Avi-tagged Cdc37 with GSTCDK4 and GSTCDK6, which were presented unpartnered, in a binary complex with cyclin D1 or cyclin D3, or in a ternary complex with cyclin D1 or D3 and p21M, or p27M. As shown previously, Cdc37 binds to monomeric CDK4 (Figures 4C and 4E, blue lines) and effectively displaces cyclin D1 or cyclin D3 (Figures 4C and 4E, red lines). However, addition of p21M or p27M to CDK4-cyclin D3 (Figure 4E, magenta and green lines, respectively) or addition of p27M to CDK4-cyclin D1 (Figure 4C, green line) prevent Cdc37 from binding, suggesting that cyclin D isoforms and the CIP/KIP CKIs cooperate to generate a stable ternary CDK4 complex that is resistant to displacement by Cdc37.

As demonstrated above, Cdc37 binds to free CDK6 (Figures 4D and 4F, blue lines) and, as expected, was less effective at extracting CDK6 from complexes with cyclin D3 (Figure 4F, red line). CDK6-cyclin D3-p27M was refractory to disruption (Figure 4F, green line). Again, cyclin D1 and D3 are distinguished: cyclin D1, unlike cyclin D3 and the viral D-type cyclins, forms a complex with CDK6 that is disrupted by Cdc37 (Figure 4D, red line). Addition of p27M can stabilize it (Figure 4D, green line). These results suggest that activation of CDK4 and CDK6 by D-type cyclins can be distinguished by their requirement for the assembly function of p21CIP1 or p27KIP1. We hypothesize that the CIP/KIP proteins assist CDK4 and CDK6 to redistribute from Cdc37 complexes into stable complexes with cyclin D1. Formation of complexes of CDK6 with cyclin D3 and the viral D-type cyclins, by contrast, are less dependent on this mechanism.

DISCUSSION

Protein kinases differ in their apparent affinity for Cdc37, and it has been hypothesized that the strength of this interaction reflects the inherent stability of the client kinase fold (Taipale et al., 2012) (Keramisanou et al., 2016). However, the simple reversibility of the interaction with Cdc37 *in vitro* and the ability of this interaction to be inhibited by small-molecule ATP-competitive kinase inhibitors (Polier et al., 2013) strongly suggest that recruitment to the Cdc37-Hsp90 system is more about regulation than protein folding (Keramisanou et al., 2016; Verba et al., 2016). Client kinases recruited to Cdc37-Hsp90 complexes are thought to undergo repeated cycles of release and recapture driven by the Hsp90 ATPase-coupled chaperone cycle until they escape the cycle through achieving an active state and/or by finding an appropriate partner (Prodromou and Pearl, 2014). Alternatively, when the chaperone ATPase cycle is blocked, the kinase becomes ubiquitylated and subsequently degraded by the proteasome (Butler et al., 2015; Krukenberg et al., 2011; Neckers and Workman, 2012). We have shown that ATP-competitive drugs targeting CDK4/6 have a shared mechanism of action with small-molecule inhibitors of other Hsp90-dependent kinases (Polier et al., 2013) and block Cdc37 binding to deprive CDK4 and CDK6 of access to the Hsp90 chaperone system. It is yet to be determined whether enhanced CDK4/6 degradation through the proteasome system may also be an unintended but therapeutically valuable consequence of the action of CDK4/6 inhibitors or whether alternative dysregulated pathways emerge (Paternot et al., 2014; Polier et al., 2013).

The central hypothesis of this paper is that much of the behavior of CDK4/6 in respect of their interactions with Cdc37-Hsp90 can be explained by thermodynamic partitioning between chaperoned (in complex with Cdc37 with or without Hsp90), activated (in complex with cyclins), and inhibited (in complex with INK) complexes (Figure 5). CDK4 and CDK6 can reversibly associate with Cdc37 in a state that is poised for displacement by CDK regulators. The recently published structure of a CDK4-Cdc37-Hsp90 complex supports this model and reveals, at least for CDK4, that the poised state is one in which the kinase is considerably unfolded (Verba et al., 2016). As a prediction of this structural model, it was proposed that rising cyclin D concentrations could provide sufficient stabilization energy so that unfolded CDK4 would be displaced from Cdc37 to form a folded CDK4-cyclin D complex. Our results extend this model to show that CDK4 and CDK6 are distinguished as Cdc37 clients by their differing affinities and that differences in the affinities of CDK regulators (INKs versus cyclin D1 versus cyclin D3) for CDK4 and CDK6 have the potential to affect CDK4 and CDK6 displacement from Cdc37 to create a network of finely tuned signaling interactions to regulate CDK4 and CDK6 activity.

We demonstrate that the different authentic INK proteins bind tightly to CDK4 and CDK6 and are highly effective at displacing

OPEN
ACCESS
CellPress

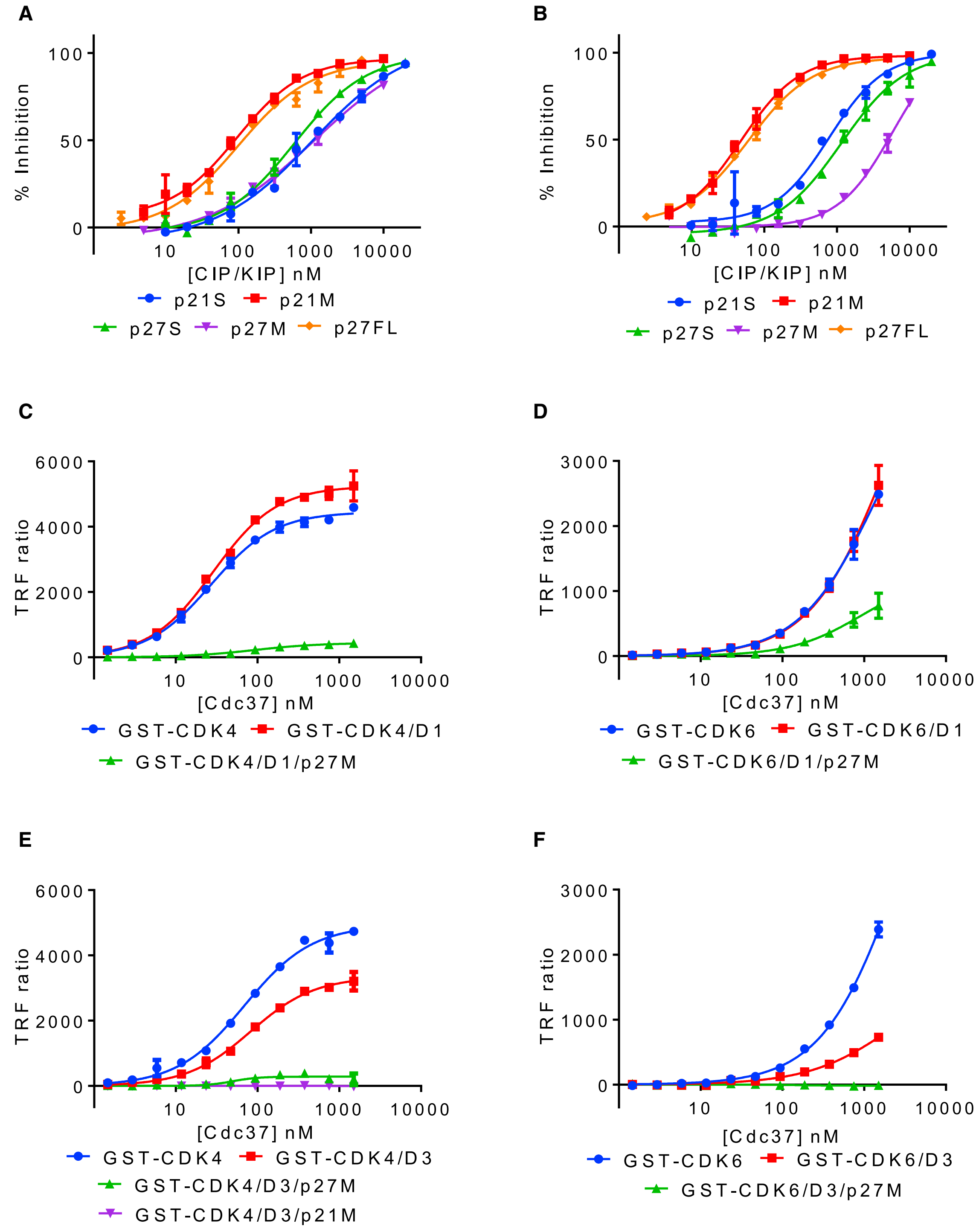

Figure 4. p21CIP1 and p27KIP1 Cooperate with Cyclin D to Prevent Cdc37 Association with CDK4 and CDK6

(A and B) p27KIP1$_{FL}$ (p27FL), p21CIP1$_{S/M}$ (p21S, p21M), and p27KIP1$_{S/M}$ (p27S, p27M) can displace CDK4 (A) or CDK6 (B) from Cdc37.

(C and D) Cdc37 binds to CDK4 (C) or CDK6 (D) (blue curves) and can effectively displace cyclin D1 (red curves). Upon addition of excess Cdc37, the ternary CDK-cyclin D1-p27KIP1$_M$ complexes do not redistribute to form CDK-Cdc37 (green curves).

(E) Cdc37 binds to CDK4 (blue) and can effectively displace cyclin D3 bound to CDK4 (red). Upon addition of excess Cdc37, ternary complexes of CDK4-cyclin D3-p27KIP1$_M$ or p21CIP1$_M$ do not redistribute to form CDK4-Cdc37 (green and magenta curves, respectively).

(F) In contrast, CDK6 binds to Cdc37 (blue curve) but cannot be displaced from cyclin D3 by Cdc37 (red curve).

All HTRF measurements were carried out in duplicate and repeated. The error bars indicate SD.

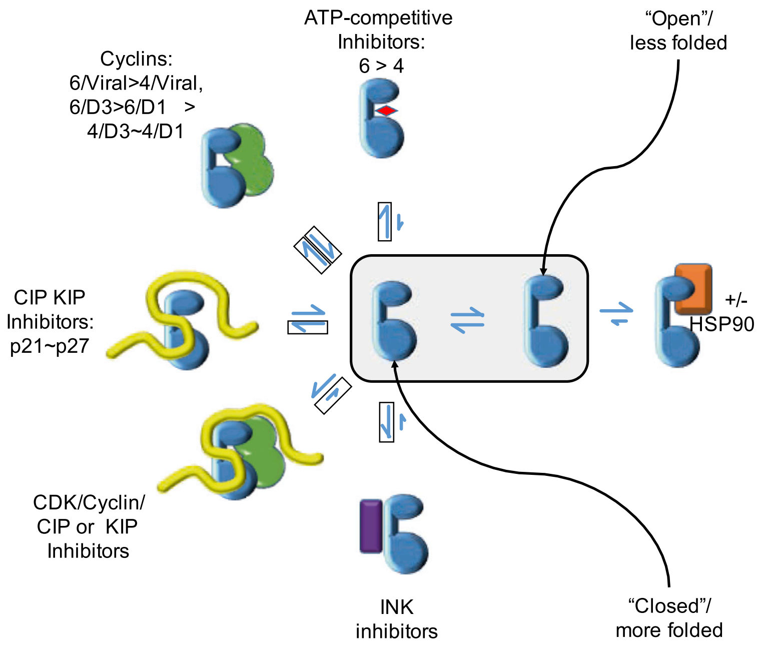

Figure 5. Exchange of CDKs into and out of Complexes Containing Cdc37

The experiments reconstitute the redistribution of CDKs 4 and 6 (blue) between complexes that contain Cdc37 (coral) and complexes that contain other binding partners drawn from ATP-competitive inhibitors (red), cyclins (green), CIP/KIP family CKIs (yellow), and INK family CKIs (purple). Boxed half arrows indicate the half reactions of these equilibria that have been reconstituted in the present study. Smaller arrows in the "reverse" direction (i.e., toward complexes that contain Cdc37) indicate reactions where the competitor appears to bind to CDKs substantially more tightly than does Cdc37. Partitioning between the different partnered states is presumed to proceed via unbound states of the kinase, which exist in an equilibrium between less folded "open" states (which preferentially bind to the chaperone system) and more folded "closed" states (which preferentially bind to the other partners).

CDK4 or CDK6 from Cdc37-Hsp90β. We also show that clinically relevant INK mutants can be selected in cancer cells that inactivate CDK4 and CDK6 through different mechanisms and, as a result, also show differing abilities to displace Cdc37 bound to CDK4 or CDK6.

The behavior of p16D84N and p16D108N, both as CDK4/6 binding proteins and as partners to which the Cdc37-Hsp90 chaperone system will relinquish their CDK4 or CDK6 clients, can be rationalized, respectively, by their location at the INK-CDK interface (D84N) and effect on INK stability (D108N). p16D84N has previously been reported to inhibit CDK4-cyclin D1 kinase activity as effectively as wild-type p16INK4a and to be modestly less effective at inhibiting G1 progression (Koh et al., 1995). Taken together, these results suggest that p16INK4a mutations can exert their effects by altering the equilibrium distribution of p16INK4a between multiple CDK4- and CDK6-containing complexes.

Mutations to p16INK4a residue M53 showed unexpected behavior, distinguishing CDK4 and CDK6. This mutant is equivalent to wild-type p16INK4a in its affinity for CDK6 but displays an enhanced k_{off} rate toward CDK4, resulting in a $>10^6$-fold reduction in the strength of the interaction. As a result, p16M53I is as effective as authentic p16INK4a in its ability to displace CDK6 from a preformed CDK6-Cdc37 complex but has a much reduced ability to displace CDK4 from Cdc37. These results suggest that the disease-associated activity of this mutant may be manifested primarily through its activity toward CDK4. This behavior has also been reported for another p16INK4a mutant also identified in familial melanoma in which R24 is mutated to a proline (Jones et al., 2007).

Cyclins, however, display a range of efficiencies. As expected, this spectrum of cyclin D activity favors CDK6-cyclin complexes because CDK6 is a weaker Cdc37-Hsp90 client. In the presence of viral cyclins or cyclin D3, CDK6 partitions from a chaperoned into a cyclin-bound complex. In the presence of cyclin D1, however, we observed different behavior. Specifically, we found that CDK6 spontaneously redistributes from cyclin D1 into a CDK6-Cdc37-Hsp90 complex, suggesting that the affinity of cyclin D1 for CDK6 is not sufficient to overcome that of Cdc37-Hsp90. CDK4 however, is a strong Cdc37-Hsp90 client, and as a result, no cyclins tested were able to partition the CDK4 away from Cdc37-Hsp90. These results suggest a fine-tuning of the system to distinguish the affinities of different cyclin D isoforms toward CDK4 and CDK6. This situation changes, however, when the cyclin is partnered by either p21CIP1 or p27KIP1. Although these CKIs are inefficient at displacing Cdc37 from CDK4/6 when alone, when present with either cyclin D1 or cyclin D3, they form a stable ternary complex that is resistant to redistribution of the CDK into a Cdc37-Hsp90 complex. We have provided direct evidence that p27KIP1 can perturb the distribution of CDKs between Cdc37-containing and cyclin-containing complexes in experiments that reconstitute the reverse reaction. Because the results presented in this paper demonstrate that both forward and reverse reactions can occur spontaneously, it is reasonable to infer that inhibition of the reverse reaction is functionally equivalent to promoting the forward reaction, an equilibrium model that is consistent with an analysis of the CDK4-Cdc37-Hsp90 structure (Verba et al., 2016). We hypothesize that this behavior provides a potential molecular mechanism for the well documented activity of p21CIP1 and p27KIP1 as "assembly factors" for CDK4/6-cyclin D-containing complexes during G1.

We propose that other CDK4 and CDK6 regulators may be able to displace both CDK4 and CDK6 from Cdc37. Such a model has been proposed from cell-based studies (Sugimoto et al., 2002). Our in vitro experiments complement this approach and provide a more quantitative assessment of the different

molecular interactions involved. The differences we observe in the interplay of CDK4 and CDK6 and their partners with the components of the Hsp90 system could explain the apparently contradictory studies in mouse embryonic fibroblasts (MEFs) that have addressed the roles of CKIs in assembling CDK4 and CDK6 complexes. MEFs lacking p21CIP1 and p27KIP1 have been reported to fail to assemble CDK4/6 complexes (Cheng et al., 1999), to assemble them but to a much lower level (Sugimoto et al., 2002), or to assemble them under conditions where cyclin D levels were elevated (Bagui et al., 2003). In another study in HCT116 cells, overexpression of Cdc37 suppressed CDK4 binding to p16 but enhanced CDK4 binding to cyclin D (Zhao et al., 2004).

Taken together with our results, a unifying model is that the CIP/KIP and INK families of CDK inhibitors may each play a role in CDK4/6-cyclin D assembly. For INKs, this role would be mediated by their ability to compete with cyclins to displace CDKs from Cdc37, whereas for CIP/KIPs, the role would be through assisting cyclin D in sequestering CDKs from Cdc37. It can be hypothesized that CDK4 and CDK6 differ in their requirements for the CKIs to act as assembly factors. Our results support a model in which CDK6 is handed over from Cdc37 to form a relatively stable CDK6-cyclin D3 complex. At the other extreme, the weaker affinity of CDK4 for cyclin D1 or cyclin D3, coupled with the higher affinity of CDK4 for Cdc37, suggests a greater dependency on CKI assembly factor activity and/or a requirement for higher cyclin D concentrations before Cdc37 would hand over this CDK to its activating partner. We note that CDK4/6-cyclin D1 complexes remain relatively unstable, suggesting that they may be susceptible to continued exchange between cyclin and Cdc37 partners unless their subsequent modification acts to stabilize them. Given that cyclin D1 is the most widely expressed cyclin D in different cell types and is most frequently implicated as a cancer driver in solid tumors (Baker and Reddy, 2012), we hypothesize that, in many clinical settings, activation of CDK6 may be enhanced by the assembly function of p21CIP1 or p27KIP1. Our results further suggest that a subset of p16INK4a mutations will be most detrimental in cancer settings driven by CDK4. Finally, we note that other CDK4/6 regulators and /or CDK4/6 phosphorylation may also provide additional stabilization energy to displace CDK4 or CDK6 from Cdc37, providing multiple opportunities for the Cdc37-Hsp90 chaperone system to fine-tune CDK4 and CDK6 activities.

EXPERIMENTAL PROCEDURES

Protein Expression

Proteins were expressed in recombinant *E. coli* cells or in *Sf*9 insect cells using a recombinant baculovirus expression system (Bieniossek et al., 2012) and purified by sequential affinity and size-exclusion chromatography.

Interaction Assays

Pull-down assays were conducted at 4°C with the target protein present in a 3-fold molar excess to the bait and visualized by SDS-PAGE and subsequent staining with InstantBlue. For HTRF assays, one of the interacting partners was purified as a GST fusion, whereas the other was expressed with an Avi tag to allow subsequent biotinylation. Interactions between the partners gave rise to a fluorescent signal because of developing reagents comprising a turbium-conjugated anti-GST antibody and the fluorescent dye SAXL665 coupled to streptavidin. For competition HTRF assays, binary complexes were established with one partner present in trace concentrations and the other poised at its apparent K_d. Competitors were titrated across a range of concentrations, and their competitive binding was observed as a loss of fluorescence resonance energy transfer (FRET) signal. For SPR assays, CDK4 and CDK6 were immobilized as GST fusions on a chip via anti-GST antibody coupling and exposed to dilution series of the various ligands. K_d values were derived from fitting the binding and dissociation curves to derive k_{on} and k_{off} rates. Data were analyzed either using GraphPad Prism version 6 (GraphPad) (HTRF) or Biacore S200 evaluation software (SPR).

DSF

Thermal melting experiments to determine T_m values for wild-type and mutant p16INK4a proteins were carried out essentially as described previously (Matulis et al., 2005).

Detailed protocols for all the methods are provided in the Supplemental Experimental Procedures.

SUPPLEMENTAL INFORMATION

Supplemental Information includes Supplemental Experimental Procedures and four figures and can be found with this article online at https://doi.org/10.1016/j.celrep.2017.10.042.

AUTHOR CONTRIBUTIONS

S.T.H., M.W.P., R.M.L.M., A.W., C.P., K.L.I.M.B., and I.F. performed the experiments; J.A.E., M.E.M.N., S.R.W., and L.H.P. supervised the project and wrote the manuscript. All authors interpreted data and commented on the manuscript.

ACKNOWLEDGMENTS

We thank our colleagues for useful discussions. This work has been funded by the MRC (G0901526 and MR/N009738/1 to J.A.E. and M.E.M.N.), CRUK (C2115/A21421 to S.R.W., J.A.E., and M.E.M.N.), and a Wellcome Trust Senior Investigator Award (095605/Z/11/Z to L.H.P.).

Received: May 19, 2016
Revised: June 10, 2017
Accepted: October 11, 2017
Published: October 31, 2017

REFERENCES

Arooz, T., Yam, C.H., Siu, W.Y., Lau, A., Li, K.K., and Poon, R.Y. (2000). On the concentrations of cyclins and cyclin-dependent kinases in extracts of cultured human cells. Biochemistry *39*, 9494–9501.

Bagui, T.K., Mohapatra, S., Haura, E., and Pledger, W.J. (2003). P27Kip1 and p21Cip1 are not required for the formation of active D cyclin-cdk4 complexes. Mol. Cell. Biol. *23*, 7285–7290.

Baker, S.J., and Reddy, E.P. (2012). CDK4: A Key Player in the Cell Cycle, Development, and Cancer. Genes Cancer *3*, 658–669.

Bieniossek, C., Imasaki, T., Takagi, Y., and Berger, I. (2012). MultiBac: expanding the research toolbox for multiprotein complexes. Trends Biochem. Sci. *37*, 49–57.

Bisteau, X., Paternot, S., Colleoni, B., Ecker, K., Coulonval, K., De Groote, P., Declercq, W., Hengst, L., and Roger, P.P. (2013). CDK4 T172 phosphorylation is central in a CDK7-dependent bidirectional CDK4/CDK2 interplay mediated by p21 phosphorylation at the restriction point. PLoS Genet. *9*, e1003546.

Blain, S.W., Montalvo, E., and Massagué, J. (1997). Differential interaction of the cyclin-dependent kinase (Cdk) inhibitor p27Kip1 with cyclin A-Cdk2 and cyclin D2-Cdk4. J. Biol. Chem. *272*, 25863–25872.

Boczek, E.E., Reefschläger, L.G., Dehling, M., Struller, T.J., Häusler, E., Seidl, A., Kaila, V.R., and Buchner, J. (2015). Conformational processing of oncogenic v-Src kinase by the molecular chaperone Hsp90. Proc. Natl. Acad. Sci. USA *112*, E3189–E3198.

Brotherton, D.H., Dhanaraj, V., Wick, S., Brizuela, L., Domaille, P.J., Volyanik, E., Xu, X., Parisini, E., Smith, B.O., Archer, S.J., et al. (1998). Crystal structure of the complex of the cyclin D-dependent kinase Cdk6 bound to the cell-cycle inhibitor p19INK4d. Nature *395*, 244–250.

Butler, L.M., Ferraldeschi, R., Armstrong, H.K., Centenera, M.M., and Workman, P. (2015). Maximizing the Therapeutic Potential of HSP90 Inhibitors. Mol. Cancer Res. *13*, 1445–1451.

Cheng, M., Olivier, P., Diehl, J.A., Fero, M., Roussel, M.F., Roberts, J.M., and Sherr, C.J. (1999). The p21(Cip1) and p27(Kip1) CDK 'inhibitors' are essential activators of cyclin D-dependent kinases in murine fibroblasts. EMBO J. *18*, 1571–1583.

Citri, A., Harari, D., Shohat, G., Ramakrishnan, P., Gan, J., Lavi, S., Eisenstein, M., Kimchi, A., Wallach, D., Pietrokovski, S., and Yarden, Y. (2006). Hsp90 recognizes a common surface on client kinases. J. Biol. Chem. *281*, 14361–14369.

Eckl, J.M., Scherr, M.J., Freiburger, L., Daake, M.A., Sattler, M., and Richter, K. (2015). Hsp90·Cdc37 complexes with protein kinases form cooperatively with multiple distinct interaction sites. J. Biol. Chem. *290*, 30843–30854.

Gu, Y., Turck, C.W., and Morgan, D.O. (1993). Inhibition of CDK2 activity in vivo by an associated 20K regulatory subunit. Nature *366*, 707–710.

Harland, M., Meloni, R., Gruis, N., Pinney, E., Brookes, S., Spurr, N.K., Frischauf, A.M., Bataille, V., Peters, G., Cuzick, J., et al. (1997). Germline mutations of the CDKN2 gene in UK melanoma families. Hum. Mol. Genet. *6*, 2061–2067.

Hunter, T., and Pines, J. (1994). Cyclins and cancer. II: Cyclin D and CDK inhibitors come of age. Cell *79*, 573–582.

Jirawatnotai, S., Sharma, S., Michowski, W., Suktitipat, B., Geng, Y., Quackenbush, J., Elias, J.E., Gygi, S.P., Wang, Y.E., and Sicinski, P. (2014). The cyclin D1-CDK4 oncogenic interactome enables identification of potential novel oncogenes and clinical prognosis. Cell Cycle *13*, 2889–2900.

Jones, R., Ruas, M., Gregory, F., Moulin, S., Delia, D., Manoukian, S., Rowe, J., Brookes, S., and Peters, G. (2007). A CDKN2A mutation in familial melanoma that abrogates binding of p16INK4a to CDK4 but not CDK6. Cancer Res. *67*, 9134–9141.

Keramisanou, D., Aboalroub, A., Zhang, Z., Liu, W., Marshall, D., Diviney, A., Larsen, R.W., Landgraf, R., and Gelis, I. (2016). Molecular Mechanism of Protein Kinase Recognition and Sorting by the Hsp90 Kinome-Specific Cochaperone Cdc37. Mol. Cell *62*, 260–271.

Koh, J., Enders, G.H., Dynlacht, B.D., and Harlow, E. (1995). Tumour-derived p16 alleles encoding proteins defective in cell-cycle inhibition. Nature *375*, 506–510.

Krukenberg, K.A., Street, T.O., Lavery, L.A., and Agard, D.A. (2011). Conformational dynamics of the molecular chaperone Hsp90. Q. Rev. Biophys. *44*, 229–255.

LaBaer, J., Garrett, M.D., Stevenson, L.F., Slingerland, J.M., Sandhu, C., Chou, H.S., Fattaey, A., and Harlow, E. (1997). New functional activities for the p21 family of CDK inhibitors. Genes Dev. *11*, 847–862.

Lamphere, L., Fiore, F., Xu, X., Brizuela, L., Keezer, S., Sardet, C., Draetta, G.F., and Gyuris, J. (1997). Interaction between Cdc37 and Cdk4 in human cells. Oncogene *14*, 1999–2004.

Larrea, M.D., Liang, J., Da Silva, T., Hong, F., Shao, S.H., Han, K., Dumont, D., and Slingerland, J.M. (2008). Phosphorylation of p27Kip1 regulates assembly and activation of cyclin D1-Cdk4. Mol. Cell. Biol. *28*, 6462–6472.

Li, M., Lee, H., Yoon, D.W., Albrecht, J.C., Fleckenstein, B., Neipel, F., and Jung, J.U. (1997). Kaposi's sarcoma-associated herpesvirus encodes a functional cyclin. J. Virol. *71*, 1984–1991.

Matulis, D., Kranz, J.K., Salemme, F.R., and Todd, M.J. (2005). Thermodynamic stability of carbonic anhydrase: measurements of binding affinity and stoichiometry using ThermoFluor. Biochemistry *44*, 5258–5266.

Morgan, D.O. (2007). The cell cycle: principles of control (Oxford University Press).

Neckers, L., and Workman, P. (2012). Hsp90 molecular chaperone inhibitors: are we there yet? Clin. Cancer Res. *18*, 64–76.

Parry, D., Mahony, D., Wills, K., and Lees, E. (1999). Cyclin D-CDK subunit arrangement is dependent on the availability of competing INK4 and p21 class inhibitors. Mol. Cell. Biol. *19*, 1775–1783.

Paternot, S., Colleoni, B., Bisteau, X., and Roger, P.P. (2014). The CDK4/CDK6 inhibitor PD0332991 paradoxically stabilizes activated cyclin D3-CDK4/6 complexes. Cell Cycle *13*, 2879–2888.

Polier, S., Samant, R.S., Clarke, P.A., Workman, P., Prodromou, C., and Pearl, L.H. (2013). ATP-competitive inhibitors block protein kinase recruitment to the Hsp90-Cdc37 system. Nat. Chem. Biol. *9*, 307–312.

Prodromou, C., and Pearl, L.H. (2014). Structural Basis of Hsp90 Function. In Inhibitors of Molecular Chaperones as Therapeutic Agents, T.D. Machajewski and Z. Gao, eds. (London: Royal Society of Chemistry), pp. 37–64.

Ray, A., James, M.K., Larochelle, S., Fisher, R.P., and Blain, S.W. (2009). p27Kip1 inhibits cyclin D-cyclin-dependent kinase 4 by two independent modes. Mol. Cell. Biol. *29*, 986–999.

Ruas, M., Brookes, S., McDonald, N.Q., and Peters, G. (1999). Functional evaluation of tumour-specific variants of p16INK4a/CDKN2A: correlation with protein structure information. Oncogene *18*, 5423–5434.

Russo, A.A., Jeffrey, P.D., Patten, A.K., Massagué, J., and Pavletich, N.P. (1996). Crystal structure of the p27Kip1 cyclin-dependent-kinase inhibitor bound to the cyclin A-Cdk2 complex. Nature *382*, 325–331.

Russo, A.A., Tong, L., Lee, J.O., Jeffrey, P.D., and Pavletich, N.P. (1998). Structural basis for inhibition of the cyclin-dependent kinase Cdk6 by the tumour suppressor p16INK4a. Nature *395*, 237–243.

Schulze-Gahmen, U., and Kim, S.H. (2002). Structural basis for CDK6 activation by a virus-encoded cyclin. Nat. Struct. Biol. *9*, 177–181.

Sherr, C.J., and Roberts, J.M. (1999). CDK inhibitors: positive and negative regulators of G1-phase progression. Genes Dev. *13*, 1501–1512.

Sherr, C.J., Beach, D., and Shapiro, G.I. (2016). Targeting CDK4 and CDK6: From Discovery to Therapy. Cancer Discov. *6*, 353–367.

Smith, J.R., de Billy, E., Hobbs, S., Powers, M., Prodromou, C., Pearl, L., Clarke, P.A., and Workman, P. (2015). Restricting direct interaction of CDC37 with HSP90 does not compromise chaperoning of client proteins. Oncogene *34*, 15–26.

Smith-Sørensen, B., and Hovig, E. (1996). CDKN2A (p16INK4A) somatic and germline mutations. Hum. Mutat. *7*, 294–303.

Soos, T.J., Kiyokawa, H., Yan, J.S., Rubin, M.S., Giordano, A., DeBlasio, A., Bottega, S., Wong, B., Mendelsohn, J., and Koff, A. (1996). Formation of p27-CDK complexes during the human mitotic cell cycle. Cell Growth Differ. *7*, 135–146.

Stepanova, L., Leng, X., Parker, S.B., and Harper, J.W. (1996). Mammalian p50Cdc37 is a protein kinase-targeting subunit of Hsp90 that binds and stabilizes Cdk4. Genes Dev. *10*, 1491–1502.

Sugimoto, M., Martin, N., Wilks, D.P., Tamai, K., Huot, T.J., Pantoja, C., Okumura, K., Serrano, M., and Hara, E. (2002). Activation of cyclin D1-kinase in murine fibroblasts lacking both p21(Cip1) and p27(Kip1). Oncogene *21*, 8067–8074.

Swanton, C., Mann, D.J., Fleckenstein, B., Neipel, F., Peters, G., and Jones, N. (1997). Herpes viral cyclin/Cdk6 complexes evade inhibition by CDK inhibitor proteins. Nature *390*, 184–187.

Taipale, M., Krykbaeva, I., Koeva, M., Kayatekin, C., Westover, K.D., Karras, G.I., and Lindquist, S. (2012). Quantitative analysis of HSP90-client interactions reveals principles of substrate recognition. Cell *150*, 987–1001.

Vaughan, C.K., Gohlke, U., Sobott, F., Good, V.M., Ali, M.M., Prodromou, C., Robinson, C.V., Saibil, H.R., and Pearl, L.H. (2006). Structure of an Hsp90-Cdc37-Cdk4 complex. Mol. Cell *23*, 697–707.

Verba, K.A., Wang, R.Y., Arakawa, A., Liu, Y., Shirouzu, M., Yokoyama, S., and Agard, D.A. (2016). Atomic structure of Hsp90-Cdc37-Cdk4 reveals that Hsp90 traps and stabilizes an unfolded kinase. Science *352*, 1542–1547.

Xu, W., Yuan, X., Xiang, Z., Mimnaugh, E., Marcu, M., and Neckers, L. (2005). Surface charge and hydrophobicity determine ErbB2 binding to the Hsp90 chaperone complex. Nat. Struct. Mol. Biol. *12*, 120–126.

Zhang, H., Hannon, G.J., and Beach, D. (1994). p21-containing cyclin kinases exist in both active and inactive states. Genes Dev. *8*, 1750–1758.

Zhao, Q., Boschelli, F., Caplan, A.J., and Arndt, K.T. (2004). Identification of a conserved sequence motif that promotes Cdc37 and cyclin D1 binding to Cdk4. J. Biol. Chem. *279*, 12560–12564.

Cell Reports, Volume *21*

Supplemental Information

Differential Regulation of G1 CDK Complexes

by the Hsp90-Cdc37 Chaperone System

Stephen T. Hallett, Martyna W. Pastok, R. Marc L. Morgan, Anita Wittner, Katie L.I.M. Blundell, Ildiko Felletar, Stephen R. Wedge, Chrisostomos Prodromou, Martin E.M. Noble, Laurence H. Pearl, and Jane A. Endicott

Supplemental Figures

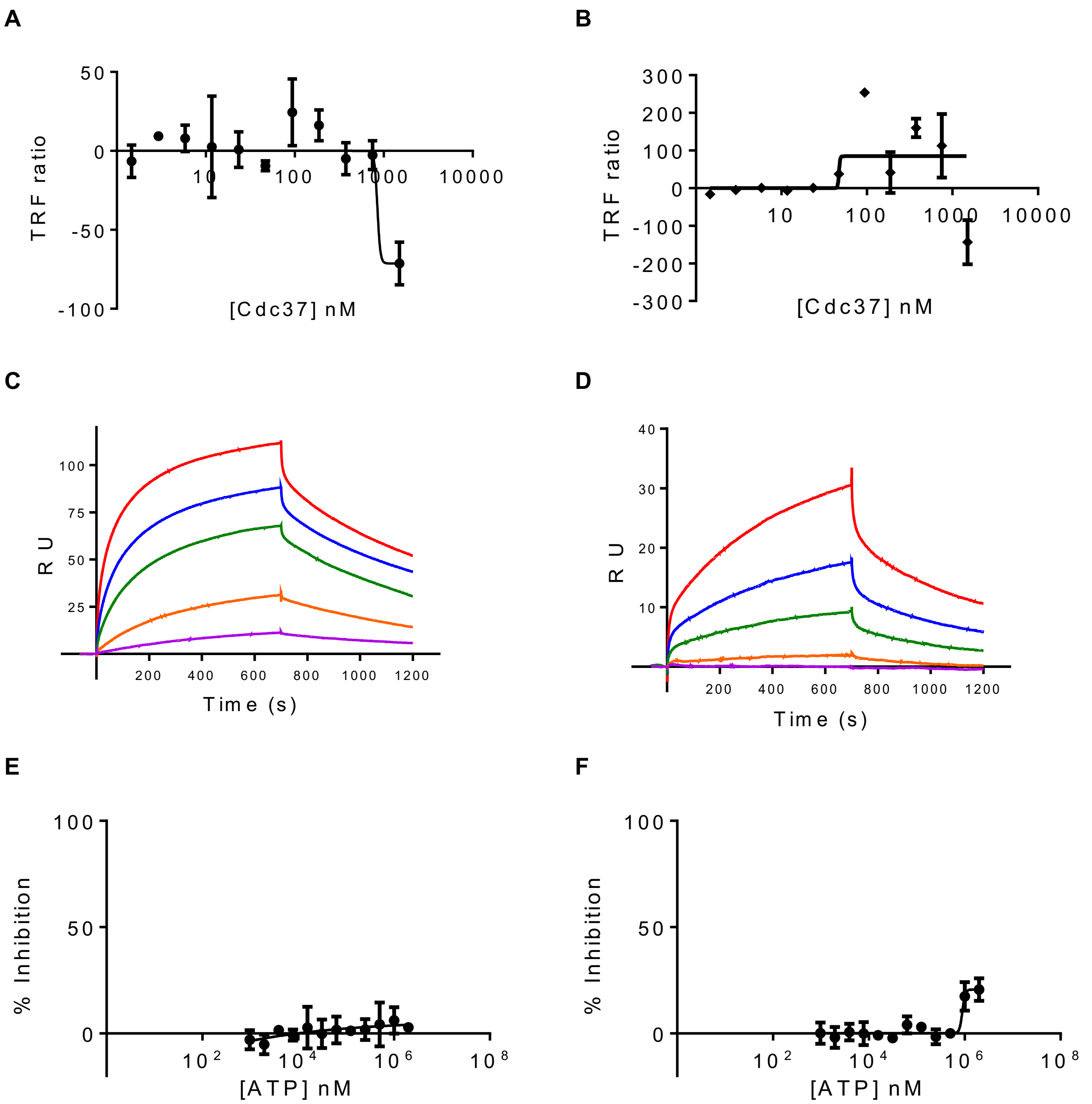

Figure S1. Related to Figure 1. Evaluation of CDK binding to Cdc37 and ATP. (A) CDK2 binding to Cdc37. The binding of full length authentic Cdc37 to CDK2 was tested by homogeneous time resolved fluorescence**.** Cdc37 does not bind to CDK2. The glutathione-S-transferase-CDK2 (GSTCDK2) concentration was 25 nM and measurements were carried out in duplicate. (**B**) Cdc37 does not bind to GST. The experiment in panel (A) was repeated replacing GSTCDK2 with GST (25 nM). In both panels, the error bars indicate standard deviation. (**C, D**) CDK4 (**C**) and CDK6 (**D**) binding to Cdc37 as measured by surface plasmon resonance. GSTCDK4 and GSTCDK6 were immobilized on the chip via anti-GST antibody coupling and the Cdc37 ligand was assayed in triplicate over a 5-point serial dilution (4000 nM, 2000 nM, 1000 nM, 250 nM and 62.5 nM). Dissociation constants (CDK4, $K_d = 347 \pm 30$ nM; and CDK6, $K_d = 7000 \pm 4500$ nM) were derived by using the Biacore S200 Evaluation Software. (**E, F**) ATP does not displace Cdc37 from CDK4 (**E**) or CDK6 (**F**). The ATP was dispensed in a two-fold dilution series starting at 2 mM. DMSO was added at 0.5% (**E**) or 0.01% (**F**). The concentrations of GSTCDK4 and GSTCDK6 were 8 nM and 6 nM respectively.

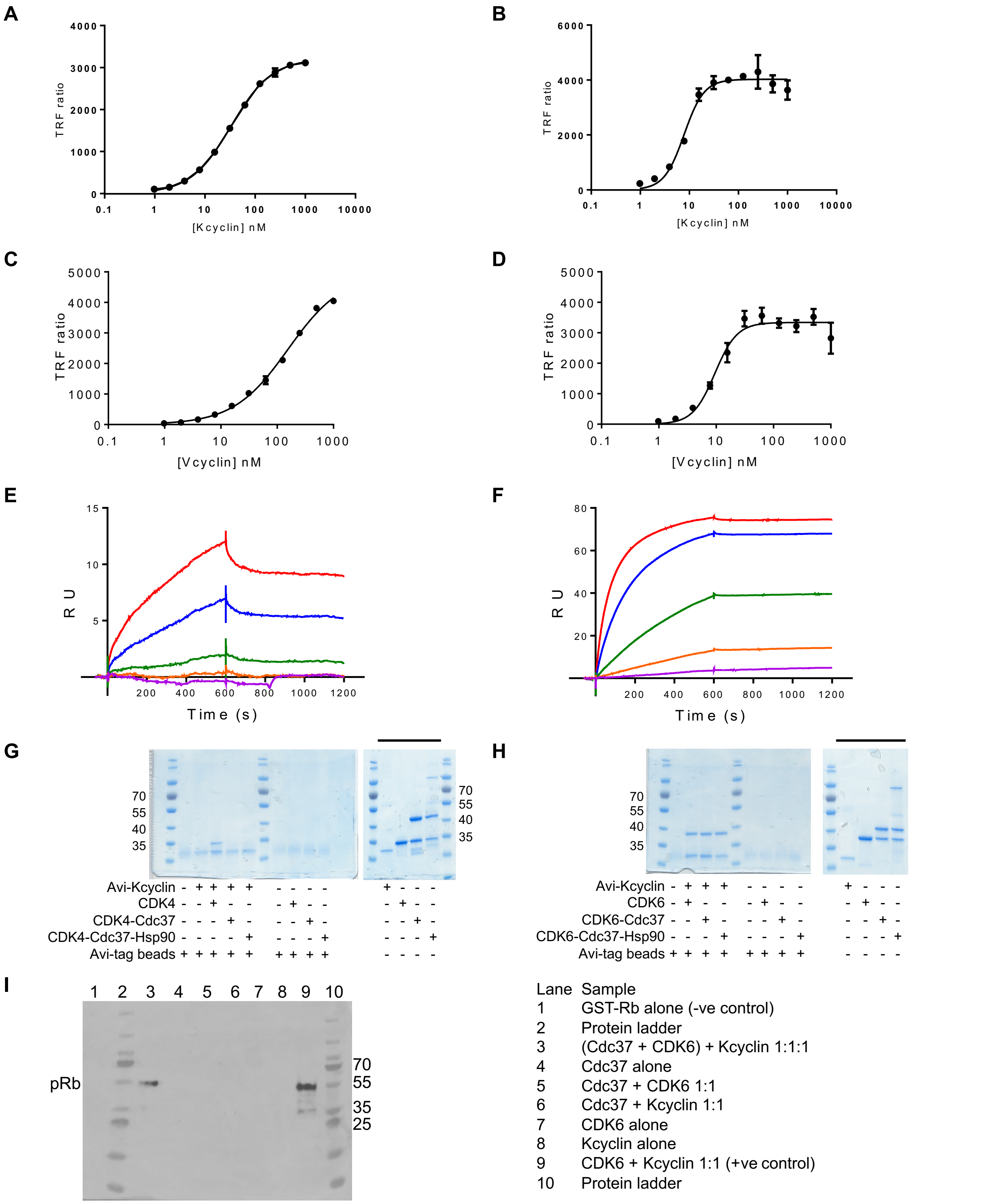

Figure S2. Related to Figure 2.

Kcyclin and Vcyclin bind to CDK4 and CDK6. (**A-D**) Homogeneous Time Resolved Fluorescence (HTRF) to measure the binding of Kcyclin (**A, B**) and Vcyclin (**C, D**) to CDK4 (**A, C**) and CDK6 (**B, D**). The concentrations of GSTCDK4 and GSTCDK6 were 10 nM. HTRF measurements were carried out in duplicate and repeated twice on two separate days. The error bars indicate standard deviation. (**E, F**) Surface Plasmon Resonance (SPR) to measure the binding of Vcyclin to CDK4 (**E**) and CDK6 (**F**). GSTCDK4 and GSTCDK6 were immobilized on the chip via anti-GST antibody coupling and the Vcyclin ligand was assayed in triplicate over a 5 point serial dilution (500 nM, 250 nM, 62.5 nM, 15.6 nM and 4 nM). Curves were evaluated using the Biacore S200 Evaluation Software. Although binding was detectable between CDK4 and Vcyclin (**E**), the data quality precluded calculation of a K_d. The dissociation constant for the interaction between CDK6 and Vcyclin ($K_d = 0.016 \pm 0.013$ nM) was derived by using the Biacore S200 Evaluation Software. **(G, H)** SDS-PAGE gels to accompany Figure 2E, F to confirm that CDK4 and CDK6 do not bind non-specifically to Avi-beads. Bars above the gels indicate inputs. Samples were visualized by InstantBlue staining. **(I)** CDK6-Kcyclin assembled following Kcyclin displacement of CDK6 from CDK6-Cdc37 is catalytically active. CDK6 activity was measured against retinoblastoma protein (pRb). A full description of the kinase assay is included in the Supplemental Experimental Procedures. Phosphorylated pRb was detected by an anti-phosphorylated S780 Ab (Abcam).

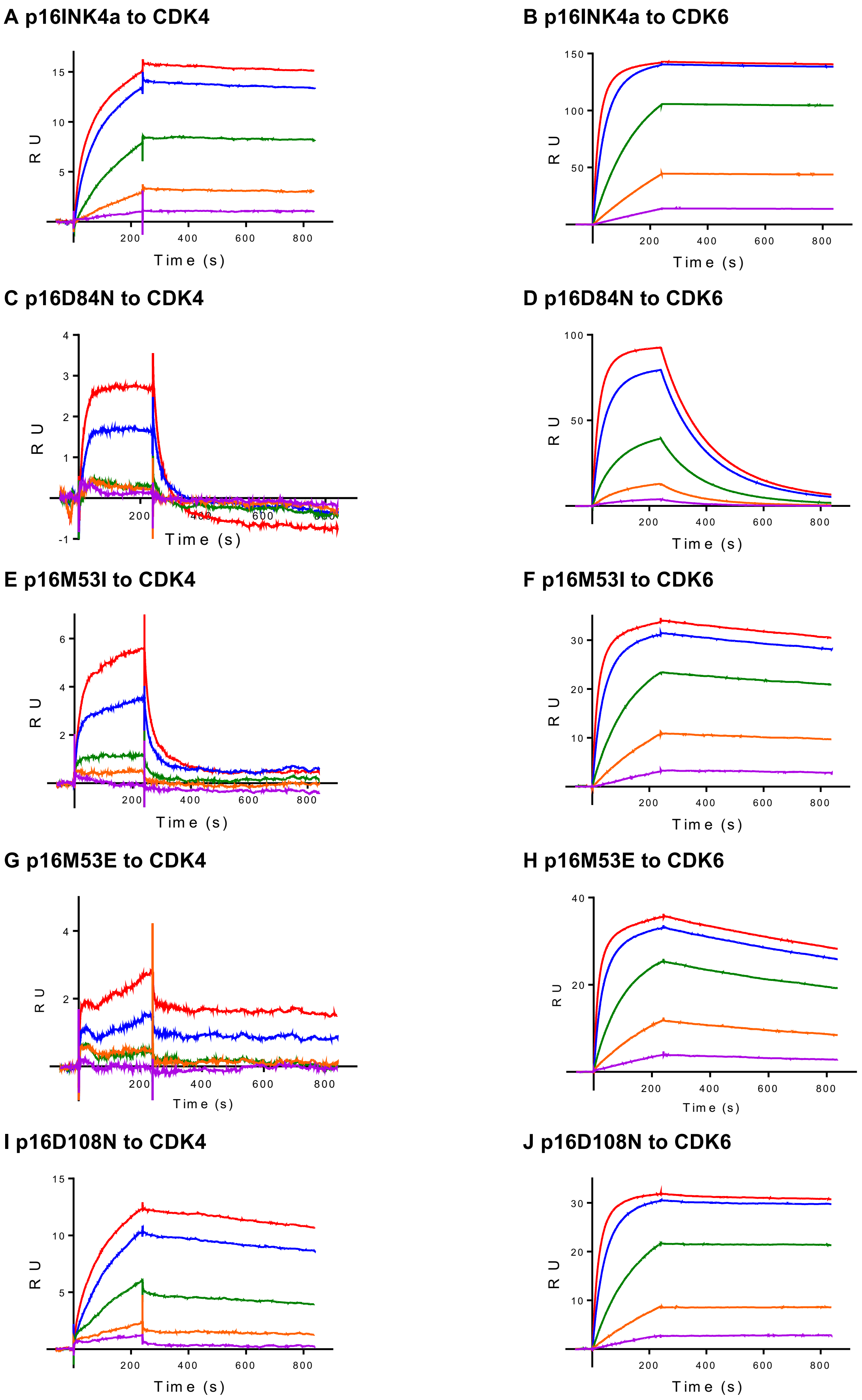

Figure S3. Related to Figure 3. p16INK4a binding to CDK4 and CDK6 measured by surface plasmon resonance. Binding of p16INK4a and p16INK4a mutants to CDK4 (**A, C, E, G, I**) and CDK6 (**B, D, F, H, J**) by SPR. GSTCDK4 or GSTCDK6 were immobilized on the chip via anti-GST antibody coupling and p16INK4a proteins were flowed over in a 5-point dilution series (200nM, 100 nM, 25 nM, 6.25 nM, and 1.56 nM). K_d values were derived using the Biacore S200 Evaluation Software and are presented in **Table 2**.

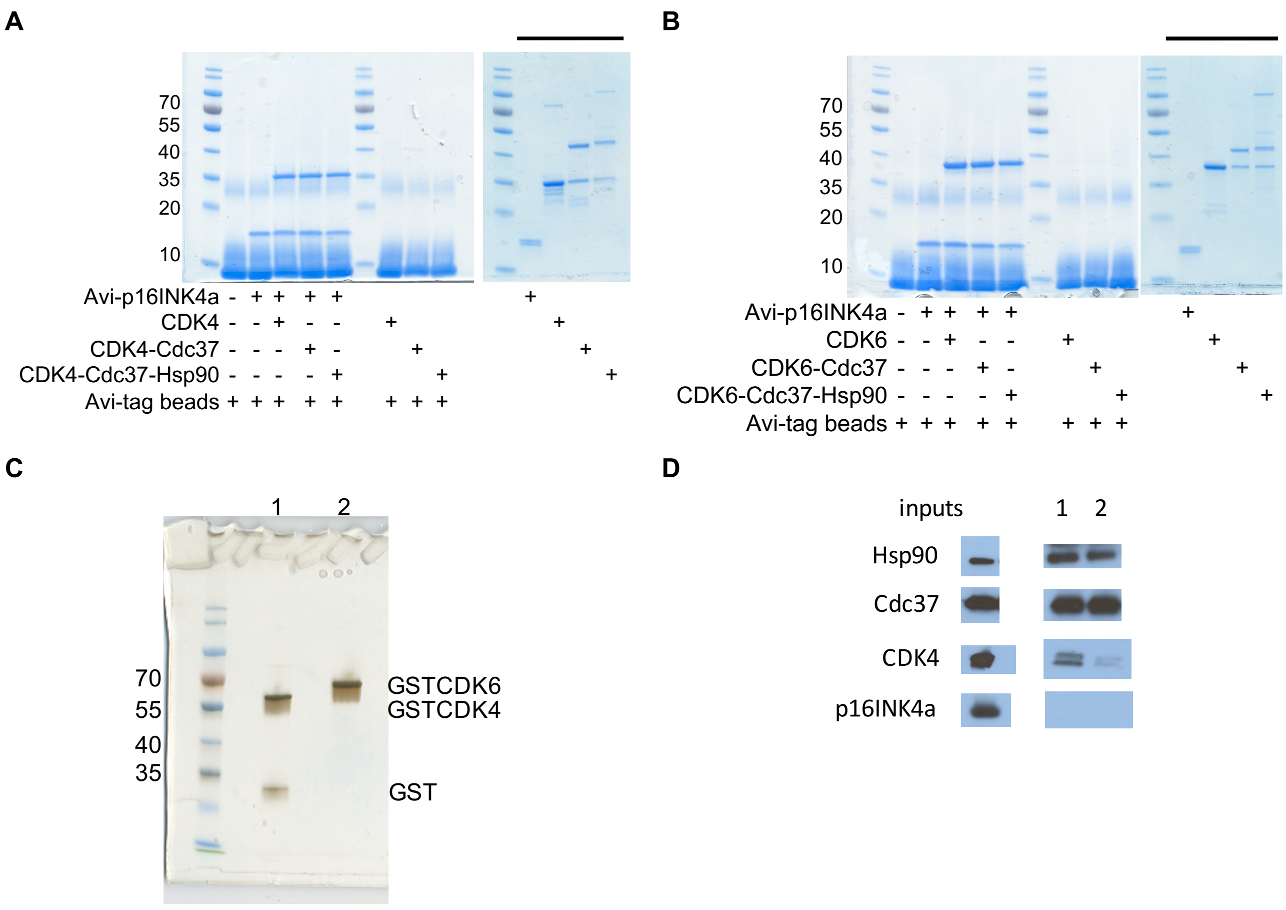

Figure S4. Related to Figure 3 and Experimental methods. Characterization of CDK4 and CDK6. (A, B) CDK4 and CDK6 do not bind non-specifically to Avi-tagged beads. Uncropped SDS-PAGE gels to accompany Figure 3 to confirm there is no non-specific binding of CDK4 and CDK6 to Avi-tag-beads. Bars above the gels indicate inputs. **(A)** CDK4, **(B)** CDK6. Samples were analyzed by SDS-PAGE and visualized by InstantBlue staining. **(C, D) Generation of GSTCDK4 and GSTCDK6 complexes from insect cells. (C)** Silver stained SDS-PAGE gel of purified GSTCDK4 and GSTCDK6. GSTCDK samples used in the HTRF assay were analyzed by SDS-PAGE and subsequent silver-staining. Lane 1, GSTCDK4; lane 2, GSTCDK6. The lower band in the GST-CDK4 sample is a small amount of contaminating GST. **(D)** The SfHsp90-Cdc37-CDK4 complex is disrupted by p16INK4a. SfHsp90-Cdc37-CDK4 purified from insect cells was incubated with buffer (lane 1) or p16INK4a (lane2) and immuno-precipitated with anti-Hsp90 antibodies. Subsequent to SDS-PAGE samples were western blotted using anti -Hsp90, -Cdc37, -CDK4 and -p16INK4a antibodies. CDK4 forms a stable complex with Hsp90 and Cdc37 (lane 1) but is displaced from Hsp90 upon addition of p16INK4a (lane 2). Cdc37 remains associated with Hsp90 upon client kinase displacement (lane 2). Input samples are 10% input.

Supplemental Experimental Procedures

Materials and reagents

Terbium-labelled anti-GST antibody and streptavidin-labelled XL665 dye were purchased from Life Technologies and Cisbio, respectively. CDK4/6 inhibitors PD0332991 (palbociclib) was purchased from Sigma Aldrich, and LY2835219 (abemaciclib) and LEE011 (ribociclib) from MedChem Express, and the Hsp90 ATP-competitive inhibitor NVP-AUY922 was supplied by Santa Cruz Biotechnology. Baculovirus expression for protein production in insect cells *Sf*9 was performed using the MultiBac™ system (Geneva-Biotech).

Protein expression

Human CDK4 and CDK6 were expressed in *Sf*9 insect cells using a recombinant baculovirus expression system (Bieniossek et al., 2012), Geneva-Biotech MultiBac™). Full length human CDK4 and CDK6 with an N-terminal glutathione-S-transferase (GST)-tag and 3C protease recognition site, and human untagged $Cdc37_{(1\text{-}348)}$ were cloned separately into the pACEBac1 acceptor vector. Human untagged full-length cyclin D1 and cyclin D3 were cloned into the pIDK donor vector. *Cre* recombinase (New England Biolabs) was used in the *Cre-Lox*P reaction of acceptor and donor vectors to generate the multigene fusion for co-expression of CDK4 or CDK6 with either cyclin D1 or cyclin D3. All constructs were verified by restriction enzyme digestion and DNA sequencing. Approximately 5-10 ng of DNA vector (either pACE-BAC-1-CDK, pACE-BAC-1-Cdc37 or an acceptor-donor fusion pACE-Bac-1-pIDK for CDK and cyclin D co-expression) was used to transform *EmBacY E. coli* cells harboring the EMBacY MultiBac™ bacmid (for constitutive expression of yellow fluorescent protein YFP). DNA sequences of interest were transferred to the bacmid via transposition into the mini Tn7 attachment site. White recombinant colonies were selected for subsequent bacmid preparation. MultiBac bacmid DNA was prepared by alkaline lysis (QIAprep Miniprep kit (Qiagen)). The final supernatant was precipitated using isopropanol (40%) and the resulting pellet was then washed twice with 70% ethanol, dried and re-suspended in sterilised water in sterile conditions. For the transfection reaction, transfection reagent (Gene Juice™ (Novagen)) was added to the re-suspended bacmid DNA and the resulting cocktail was used to infect $0.5*10^6$ *Sf9* cells seeded in 6-well plates. After 48-60 hr incubation at 27 ^oC, the supernatant was collected and positive transfection was verified by monitoring the appearance of yellow cells constitutively expressing the YFP gene and also containing the constructs of interest by fluorescence microscopy. This initial virus stock (Vo) was amplified twice and then used for protein expression.

Other proteins were expressed from recombinant *E. coli* cells. Human CDK2 was expressed as a GST fusion from the pGEX6P-1 vector backbone (GE Healthcare) as previously described (Brown et al., 1999). The full-length DNA sequences encoding herpesvirus saimiri cyclin (Vcyclin) and Kaposi's sarcoma-associated herpesvirus cyclin (Kcyclin) were synthesized (Integrated DNA Technologies). These sequences were sub-cloned into pGEX6P-1 to generate N-terminal GST fusion proteins and also further modified to include either a C-terminal Avi or FLAG tag. Mutant p16INK4a sequences were generated by site-directed mutagenesis using the QuikChange method (Agilent Technologies) or by gene synthesis (Integrated DNA Technologies) and verified by sequencing. The INK sequences were subsequently cloned into a modified pET3d vector to produce a N-terminal His_6-tagged protein that upon 3C cleavage generates the INK modified by a N-terminal Avi-tag. Full-length p27KIP1, p27KIP1, p21CIP1 and pRb fragments were expressed as GST-fusions from a pGEX6P-1 backbone. For the pull down experiments, full-length Cdc37 was modified with a N-terminal 3C protease-cleavable hexa-histidine tag following cloning into a pRSETA vector backbone. 3C cleavage generates a Cdc37 construct with a FLAG-tag at the N-terminus. For the HTRF experiments, Cdc37 was expressed from a modified pET3d vector to generate C-terminally Avi-tagged proteins. The Hsp90β full length construct was used in this study. This sequence was also expressed with an N-terminal 3C protease-cleavable hexa-histidine tag from a pRSETA vector backbone and for the HTRF assay was expressed from a modified pET3d vector backbone to generate an N-terminally Avi-tagged protein.

Protein purification

Insect cells were harvested after 72 hours of infection, re-suspended in 10 mM HEPES pH 7.5, 150 mM NaCl, 10% glycerol, 0.5 mM EDTA, 0.5 mM TCEP (resuspension buffer) supplemented with a protease inhibitor cocktail (Roche) and stored at -20 ^oC. Cell pellets were sonicated on ice followed by centrifugation at 48254xg for 1 hr at 4 ^oC. The filtered supernatant was incubated with 1 ml of glutathione Sepharose (GE Healthcare) for 3 hr on a rocker at 4 ^oC. The Sepharose resin was then loaded into a column and exhaustively washed with 30 ml of resuspension buffer. Bound proteins were eluted by 20 mM gluthathione at pH 8.0, cleaved overnight with 3C protease (1:50 w/w) and then further purified using size exclusion chromatography (SEC) (HiLoad 16/60 Superdex 75 pg for proteins smaller than ~60 kDa or a 200 pg for proteins or complexes larger than ~60 kDa). To ensure removal of any contaminating GST dimer, appropriate SEC fractions were pooled, incubated with glutathione Sepharose and then concentrated.

Human CDK2, INKs, p21CIP1, p27KIP1, Cdc37, Hsp90β and viral cyclins were expressed in *E. coli* strain BL21STAR(DE3) (Life Technologies) (CDK2, INKs, p21CIP1, p27KIP1, Cdc37, and viral cyclins) or Rosetta (DE3) (Cdc37 and Hsp90β). Briefly, recombinant *E. coli* BL21STAR(DE3) cells were grown at 30 ^oC in 2xYT medium till OD_{600}~0.7-0.8, induced at 18 ^oC by adding 0.5 mM IPTG and further incubated for 16 hr. Alternatively recombinant *E. coli* Rosetta (DE3) cells (Merck Millipore) expressing Cdc37 or Hsp90β were grown at 37 ^oC in LB medium till the OD_{600} reached ~0.2 and 0.6 respectively. The cells were then induced at 18 ^oC by adding 0.5 mM IPTG and incubated for 16 hr. GST-tagged proteins were first purified by an affinity chromatography step, followed by 3C protease cleavage to remove the GST tag and then subsequent SEC. If required, appropriate SEC fractions were pooled and re-applied to a glutathione column to remove any contaminating GST and the flow-through collected. His_6-tagged proteins were purified using Ni-NTA His-Trap (GE Healthcare), cleaved and further purified using sequential SEC and ion

exchange chromatography (HiTrap Q, GE Healthcare). Protein concentrations were determined by NanoDrop2000 UV-Vis Spectrophotometer (Thermo Scientific) or using a Shimadzu UV1800 spectrophotometer. A representative SDS-polyacrylamide gel stained with silver is shown in Figure S4 to illustrate the purity of the GSTCDK4 and GSTCDK6 post purification.

Protein biotinylation
Avi-tagged proteins (Cdc37, p16INK4a, p16INK4a mutants, Kcyclin and Vcyclin) were biotinylated to provide an affinity tag for the streptavidin-tagged XL665 dye. A substantial amount of biotinylated protein was purified from the recombinant *E. coli* cells as a result of endogenous BirA activity. Proteins were further modified by incubating 40 µM of the Avi-tagged protein with 10 µg BirA in biotinylation buffer (50 mM Bicine pH 8.3, 10 mM ATP, 10 mM MgOAc, 50 µM d-biotin) at 30°C for 60 minutes. Protein was then buffer exchanged into HTRF buffer A (50 mM HEPES pH 7.5, 100 mM NaCl, 0.1 mg/ml BSA, 1 mM DTT) using a PD-10 desalting column (GE Healthcare). The extent of protein biotinylation was monitored by pull downs using High Capacity NeutrAvidin® Agarose Resin (Thermo Scientific Pierce).

Pull-down assays
For the pull down assays presented in Figure 2C, D, 6.3 µg (0.12 nmol) of FLAG-tagged Cdc37 was incubated for 1 hr at 4 ^{o}C with 25 µl anti-FLAG M2 agarose beads in a total volume of 60 µl in 20 mM Tris pH 7.5, 150 mM NaCl, 5 mM Na_2MoO_4, 0.5 mM EDTA, 0.5 mM TCEP, and 0.5% Igepal. Using this buffer non-specific protein binding to the beads was minimized. A 3-fold molar excess (0.36 nmol) of the second protein (CDK or CDK-cyclin D was then added and incubation continued for a further hour at 4 ^{o}C. Each pull down was washed three times with 200 µl of buffer and then 15 µl of SDS-PAGE sample buffer was added. Boiled and spun down samples were subjected to SDS-PAGE and proteins visualized by InstantBlue staining.

Pull-down assays of chaperone complexes assembled in insect cells
For the pull down assays presented in Figure 2E, F, Figure 3C, D and Figure S2G and H, chaperone complexes containing $Hsp90_{Sf9}$-Cdc37-CDK4 or $Hsp90_{Sf9}$-Cdc37-CDK6 were generated by co-expressing human GSTCDK4 or GSTCDK6 with human untagged $Cdc37_{(1\text{-}348)}$ in *Sf*9 insect cells using the baculovirus expression system as described above. Complexes were purified using the GST tag on the CDK as described in Vaughan *et al.* (Vaughan et al., 2006). Chaperone complexes were separated by SEC (Superdex 200 16/60 (GE Healthcare)). Fractions containing $Hsp90_{Sf9}$-Cdc37-CDK4 or $Hsp90_{Sf9}$-Cdc37-CDK6 were pooled and concentrated. For pull downs, 4.8 µg (0.150 nmol) of biotinylated C-terminally Avi-tagged viral cyclin K (D-type cyclin) or p16INK4a was incubated for 1 hr at 4 ^{o}C with 25 µl NeutrAvidin® Agarose Resin in a toal volume of 60 µl (Thermo Scientific Pierce) in 20 mM Tris pH 7.5, 150 mM NaCl, 5 mM Na_2MoO_4, 0.5 mM EDTA, 0.5 mM TCEP and 0.5% Igepal. A 3-fold molar excess of chaperone complex purified from insect cells was added and incubated for a further hour at 4 ^{o}C. Each pull down was washed as described above and boiled prior to separation by SDS-PAGE. Proteins were visualized by InstantBlue staining.

pRb kinase assay
To assemble the various complexes, 15 µg of Cdc37 was mixed with a molar equivalent of CDK6, and incubated for 1hour at 4 ^{o}C. Vcyclin was then added to appropriate samples at a 1:1 molar ratio CDK6:Vcyclin and incubated for a further hour at 4 ^{o}C. Vcyclin complexes with Cdc37 or CDK6 were generated by mixing proteins at equivalent 1:1 molar ratios. During incubation steps, the samples were manually mixed half-way through. 200 µl of glutathione bead slurry was washed in pRb assay buffer (50 mM HEPES, pH7.5, 10 mM $MgCl_2$, 1 mM DTT, 0.1 mM orthovandate, 1 mM NaF, 0.1 mM EDTA) three times and then 1 mg of GST-pRb encoding pRb residues 379-928 (Kato et al., 1993) was added and incubated for 30 mins at 4 ^{o}C. The beads were pelleted, the excess supernatant removed and then re-suspended to 200 ul volume. 20 µl of GST-pRb–bead slurry was added to each reaction tube, and the volume made up to 100 µl using pRb assay buffer. The kinase reactions were initiated by the addition of ATP to a final concentration of 2 mM, then allowed to proceed for 30 mins at 30 ^{o}C with gentle agitation. The reactions were stopped by addition of 2 x LDS buffer and analysed by SDS-PAGE and subsequent western blotting using an anti-phosphorylated S780 antibody (Abcam, ab173289).

Homogenous time-resolved fluorescence (HTRF)
(a) Direct binding format: In the direct binding assay format glutathione-S-transferase (GST)-tagged cyclin-dependent kinase (CDK) is incubated with biotinylated Avi-tagged protein of interest to form a GSTCDK-protein complex. The complex is then incubated with a Tb-labeled anti-GST antibody and streptavidin-tagged XL665 dye. Formation of a complex brings the Tb and XL665 into proximity so that excitation of the Tb results in emission from the XL665 dye as a result of Förster resonance energy transfer (FRET) between the two probes. To optimize the assay for direct binding measurements the biotinylated protein of interest was initially titrated, over 11 serial dilution points and an additional buffer blank point, against 5 fixed concentrations of either GSTCDK4 or GSTCDK6 (100 nM, 50 nM, 25 nM, 10 nM and 5 nM). Subsequently experiments to assay the various GSTCDK complexes described in Figures 1B; 4C-F; and Figure S2A-D were carried out at a fixed GSTCDK concentration of 10 nM. The concentration of GSTCDK2 and GST used in Figure S1A, B was 25 nM. Concentrations of GSTCDK and the binding protein of interest were prepared in HTRF buffer A (50 mM HEPES, 100 mM NaCl, 1 mM DTT and 0.1 mg/ml BSA) and incubated together for 60 minutes at 4°C or on a shaker at room temperature. 5 nM Tb labeled anti-GST antibody and SAXL665 at 1/8th the concentration of the biotinylated protein, were prepared in HTRF buffer B (50 mM HEPES, 100 mM NaCl and 0.1 mg/ml BSA) and added to each well. The plate was incubated for a further 120 minutes at 4°C, before being scanned. Samples were excited using a wavelength of 337 nm and emission spectra measured at 620 nm and 665 nm (PHERAstar FS (BMG LABTECH)). Binding curves were plotted using GraphPad Prism 6 from which the K_ds were

determined. The curves shown are representative binding curves from at least two runs with each mutant carried out on separate days.

(b) Competition mode: 8 nM GSTCDK4 and 150 nM C-Avi Cdc37, or 6 nM GSTCDK6 and 500 nM C-Avi Cdc37 (the Cdc37 concentration was adjusted to the measured K_d for the CDK-Cdc37 interaction), were added to each well along with a serial dilution of the competitor (viral cyclins, Figure 2A, B; INKs, Figure 3A, B, E, F; CIP/KIPs, Figure 4A, B; ATP competitive inhibitors, Figure 1C, D; or ATP, Figure S1E, F). All dilutions were made using HTRF buffer A (50 mM HEPES, 100 mM NaCl, 1 mM DTT and 0.1 mg/ml BSA) and set up in duplicate. ATP-competitive inhibitor stocks were prepared either in 100% DMSO (LEE011) or water (PD0332991 and LY2835219). LEE011 titrations were carried out at a final DMSO% of 0.1%. The plate was incubated for 60 minutes at 4°C, before 0.5 nM Tb labeled anti-GST antibody and 18.75 nM of SAXL665 (for the CDK4 measurements) or 3 nM Tb labeled anti-GST antibody and 62.5 nM of SAXL665 (for the CDK6 measurements), were added to each well. Concentrations of the Tb antibody and SAXL665 were made up using HTRF buffer B (50 mM HEPES, 100 mM NaCl and 0.1 mg/ml BSA). The plate was incubated for a further 120 minutes at 4°C, before being scanned. Samples were excited using a wavelength of 337 nm and emission spectra measured at 620 nm and 665 nm (PHERAstar FS (BMG LABTECH)). Percentage inhibition graphs were plotted using GraphPad Prism 6, by comparing to a maximum signal, where no competitor is present, and a minimum signal, where no GSTCDK4 or GSTCDK6 is present.

Surface Plasmon Resonance (SPR)
All SPR experiments were performed on a Biacore S200 (GE Healthcare) at 4 °C using SPR buffer (10 mM HEPES pH 7.5, 150 mM NaCl, 3 mM EDTA and 0.05% Tween 20). Samples were centrifuged at 10000 x g for 10 minutes at 4 °C before use. 40 µg/mL GSTCDK4 or 30 µg/mL GSTCDK6 were captured on a surface of goat anti-GST antibody (GE Healthcare) at 5 µL/min for 600s. Antibody capture to the CM5 BIAcore sensor chip was via amine coupling using the standard protocol provided in the GST capture kit (GE Healthcare). Analyte solutions of p16INK4a and p16INK4a mutants at 200 nM, 100 nM, 25 nM, 6.25 nM and 1.56 nM were flowed over the bound GSTCDKs at 30 µL/min for 240s and the dissociation was measured over 600s. Analyte solution of Cdc37 at 4000 nM, 2000 nM, 1000 nM, 250 nM and 62.5 nM were flowed over for 700s and the dissociation measured at 600s. In case of viral cyclins: 500 nM, 250 nM, 62.5 nM, 15.6 nM and 4 nM for 600s and dissociation was measured at 600s. The bound GSTCDKs were removed from the antibody using 10 mM glycine pH 2.1 at 30 µL/min for 240s. The chip was regenerated by capturing fresh GSTCDKs before each analyte concentration run. GST was loaded onto a separate lane at 10 µg/mL for 120s at 5 µL/min to act as a negative control. Sensorgram readings from the GST control were subtracted from GSTCDK sensorgrams to correct for non-specific interactions and bulk effects. K_d values and dissociation rates were calculated using the Biacore S200 Evaluation Software.

Differential Scanning Fluorimetry
A protein/Sypro orange mix containing 5 µM protein and a 1:5,000 dilution of dye in DMSO (as supplied by Sigma Aldrich) was prepared just before plate setup in 10 mM HEPES pH 7.5, 150 mM NaCl, 0.5 mM EDTA and 0.5 mM TCEP. 14.5 µL of the protein/Sypro orange mix was aliquoted into 384-well plates and sealed. Each experiment was done in triplicate. Thermal melting experiments were carried out using an QuantStudio Real Time PCR machine (Applied Biosystems). The plates were first equilibrated at 25°C for 2 min in the PCR machine before starting the thermal melting experiment upon which the plates were heated at 0.05°C per second from 25 to 99°C. The fluorescence intensity was recorded using the ROX filters under continuous collection. Raw fluorescence data were extracted from the QuantStudio Real-Time PCR software and analysed using the Applied Biosystems Protein Thermal Shift software. Calculated derivative Tm values for each of the three repeats were extracted and the average values calculated.

Silver staining
3 µg of complexes GST-CDK4 or GST-CDK6 used in HTRF and SPR assays were run on 10% SDS-PAGE gel (Biorad) for 35 minutes in 1xTGS Buffer (Biorad) at 200 V. Subsequently gels were silver stained using Pierce Silver Stain Kit (ThermoScientific) using manufacturer instructions. The gel was stained until bands started appearing and stopped with 5% acetic acid for 10 minutes, followed by 2x 5 minutes washes with ultrapure water.

Western blotting
*Sf*Hsp90-Cdc37-CDK4 purified from insect cells (10 µg) was incubated with buffer or p16INK4a (20 µg) for 60 minutes on ice and immuno-precipitated with anti-Hsp90 antibodies (abcam AC88 #ab13492) overnight. Bead bound proteins were washed 3x times with Lysis/Wash buffer (ThermoFisher Scientific, #26146). Subsequent to SDS-PAGE samples were western blotted using anti -Hsp90 (Cell Signaling, #4874), -Cdc37 (ThermoFisher Scientific, C1 #MA3-029), -CDK4 (Cell signaling, CDK4 (D9G3E) #12790) and -p16INK4a (Abcam, DCS50.1, #ab16123) antibodies.

Supplemental References

Bieniossek, C., Imasaki, T., Takagi, Y., and Berger, I. (2012). MultiBac: expanding the research toolbox for multiprotein complexes. Trends Biochm Sci *37*, 49-57.

Brown, N.R., Noble, M.E., Lawrie, A.M., Morris, M.C., Tunnah, P., Divita, G., Johnson, L.N., and Endicott, J.A. (1999). Effects of phosphorylation of threonine 160 on cyclin-dependent kinase 2 structure and activity. J Biol Chem *274*, 8746-8756.

Kato, J., Matsushime, H., Hiebert, S.W., Ewen, M.E., and Sherr, C.J. (1993). Direct binding of cyclin D to the retinoblastoma gene product (pRb) and pRb phosphorylation by the cyclin D-dependent kinase CDK4. Genes Dev *7*, 331-342.

Vaughan, C.K., Gohlke, U., Sobott, F., Good, V.M., Ali, M.M., Prodromou, C., Robinson, C.V., Saibil, H.R., and Pearl, L.H. (2006). Structure of an Hsp90-Cdc37-Cdk4 complex. Mol Cell *23*, 697-707.
